# Metformin inhibits lithocholic acid-induced interleukin 8 upregulation in colorectal cancer cells by suppressing ROS production and NF-kB activity

**DOI:** 10.1038/s41598-019-38778-2

**Published:** 2019-02-14

**Authors:** Thi Thinh Nguyen, Trong Thuan Ung, Shinan Li, Sen Lian, Yong Xia, Sun Young Park, Young Do Jung

**Affiliations:** 1 Department of Biochemistry, Chonnam National University Medical School, Seoyang Ro 264, Hwasun, Jeonnam 58138 Korea; 2 Department of Biochemistry and Molecular Biology, School of Basic Medical Sciences, Southern Medical University, Guangzhou, 510515 Guangdong, China; 3 Department of Urology, New York University School of Medicine, New York, NY 10010 USA

## Abstract

Metformin, an inexpensive, well-tolerated oral agent that is a commonly used first-line treatment for type 2 diabetes, has become the focus of intense research as a potential anticancer agent. In this study, we describe the inhibitory effect of metformin in interleukin 8 (IL-8) upregulation by lithocholic acid (LCA) in HCT116 colorectal cancer (CRC) cells. Pharmacological inhibition studies indicated that reactive oxygen species (ROS) were involved in LCA-induced IL-8 upregulation through activation of the transcription factor NF-κB. Metformin was demonstrated to block LCA-stimulated ROS production, in turn suppressing NF-κB signaling that was critical for IL-8 upregulation. An NADPH oxidase assay proved that the inhibitory effect of metformin on ROS production was derived from its strong suppression of NADPH oxidase, a key producer of ROS in cells. Compared with conditioned media (CM) derived from HCT116 cells treated with LCA, CM derived from HCT116 cells pretreated with metformin and then treated with LCA lost all stimulatory effect on endothelial cell proliferation and tubelike formation. In conclusion, metformin inhibited NADPH oxidase, which in turn suppressed ROS production and NF-κB activation to prevent IL-8 upregulation stimulated by LCA; this prevention thus obstructed endothelial cell proliferation and tubelike formation.

## Introduction

Metformin (1,1-dimethylbiguanide hydrochloride) is a biguanide derivative that belongs to a class of oral hypoglycemic agents. In the liver, metformin inhibits hepatic glucose production, resulting in enhanced blood glucose control and fewer complications associated with diabetes^1,2^. Metformin has been used worldwide not only as a first-line anti-diabetes medication but also for treatment of polycystic ovarian syndrome, metabolic syndrome, nonalcoholic fatty liver disease, and other conditions^3^.

In the past decade, metformin has become the focus of intense research as a potential anticancer agent. The first report, by Evans *et al*. on 923 cases of cancer in 11,876 newly diagnosed type 2 diabetic patients, revealed that the overall cancer incidence was lower in diabetic patients treated with metformin than in patients treated with other drugs^4^. Since this study, an increasing number of retrospective analyses have been performed. Authors of these studies reported similar trends of metformin’s effects in reducing the incidence and mortality of cancer^5^ and the occurrence of metastatic disease^6^ and in improving chemotherapeutic outcomes^7^.

Along with abundant epidemiological proof, prospective and ongoing clinical trials are also being performed to investigate the safety and the efficacy of metformin in cancer patients, with the majority of studies focusing on breast cancer. In one study, Hadad *et al*. administered metformin to nondiabetic breast cancer patients before surgery. Although there was no quantifiable change in tumor size after 2–3 weeks of metformin treatment, analysis of the tumor-derived biopsies revealed decreased insulin levels and a decrease in Ki67 staining, a marker of proliferation, indicating possible biological effects on tumor tissues^8^. Recently, a study was performed with 39 newly diagnosed, untreated, nondiabetic breast cancer patients in which the patients were administered 500 mg metformin for an average of 18 days. Not only did their body mass index, weight, and homeostatic model assessment index decrease significantly, the Ki67 staining in invasive tumor tissue decreased from 36.5% to 33.5% and dUTP nick end labeling staining increased from 0.56 to 1.05, suggesting that metformin has beneficial cancer-inhibiting effects^9^.

Although there is substantial epidemiological and clinical evidence for metformin’s efficacy in cancer prevention, the molecular mechanism of its action on cancer is not fully understood. Researchers have proposed two ways that metformin could affect tumors. First, insulin is known to prompt cancer cells to divide, so the slower rate of tumor growth could just be a side effect of metformin reducing the amount of insulin in the blood. Alternatively, metformin could target cancer cells more directly by mainly involving AMP-activated protein kinase (AMPK). Through activating AMPK, metformin reduces mammalian target of rapamycin complex 1 (mTORC1), a pivotal pathway that controls the growth, proliferation, and metabolism of cancer cells^10,11^. AMPK is also involved in p53-mediated cell cycle arrest induced by metformin^12^. Buzzai and colleagues demonstrated that in colorectal cell lines, glucose deprivation induced p53-dependent autophagy by activating AMPK in response to metformin^13^. In addition, metformin was documented to reduce chronic inflammatory responses at least partially by inhibiting the production of tumor necrosis factor alpha, preventing tumor development^14^. Production of ROS was also found to be a target of metformin in its anticancer mechanism by inhibiting mitochondrial complex I, the cellular source of ROS production, to reduce DNA damage and mutagenesis^15^.

Colorectal cancer (CRC) is one of the most common cancers and is substantially documented to be effectively treated with metformin. One meta-analysis of 37 studies with 1,535,635 total participants published in 2013 revealed metformin-specific reduction rates for the overall incidence of liver, pancreatic, colorectal, and breast cancers among diabetic type II patients of 78%, 46%, 23%, and 6%, respectively^16^. Another meta-analysis showed that metformin was associated with increased overall survival: a high dose of metformin lowered CRC-specific mortality^17^. Along with these findings, researchers have published scientific reports to attempt to determine how metformin can suppress CRC tumors. It is assumed that metformin exhibits anti-angiogenic and antiproliferative effects on CRC cells^18^. Inhibiting the mTOR pathway, an important signaling pathway in protein translation and cell proliferation, and enhancing the activity of the tumor suppressor protein p53 were demonstrated to be involved in this inhibitory mechanism of metformin^13,19^.

Lithocholic acid (LCA), a secondary bile acid, along with deoxycholic acid was proved as an endogenous CRC promoter^20,21^. Studies have suggested that LCA damages the epithelial lining of the gastrointestinal tract through production of reactive oxygen species, which results in resistance to apoptotic cell death and increased cell proliferation in gastrointestinal tract compartments^22^. LCA induction of cancer stemness has been evidenced by the increased proportion of cancer stem cells (CSCs), elevated levels of CSC markers and epithelial mesenchymal transition markers, and increased colonosphere formation^23^. LCA has also been proved to decrease the expression of HLA antigen on the surface of colon cancer cells, which helps tumor cells escape immune surveillance^24^. Additionally, LCA and other bile acids induce a marked rise in the expression level of matrix metalloproteinase genes^25–27^, leading to enhanced cancer cell invasion and tumor metastasis. Our study showed that LCA stimulated urokinase plasminogen activator receptor (uPAR), causing an increase in CRC cell invasion^20^. Additionally, in our latest study, we proved that LCA induced IL-8 expression in CRC cell lines, which in turn stimulated CRC cell angiogenesis^28^. In the current study, we describe a novel mechanism of metformin in preventing CRC tumor development by which metformin inhibited LCA-induced IL-8 upregulation in HCT116 CRC cells and obstructed the proliferation and tubelike formation of ECV304 endothelial cells.

## Results

### Metformin inhibits IL-8 upregulation stimulated by LCA

**Figure 1:**
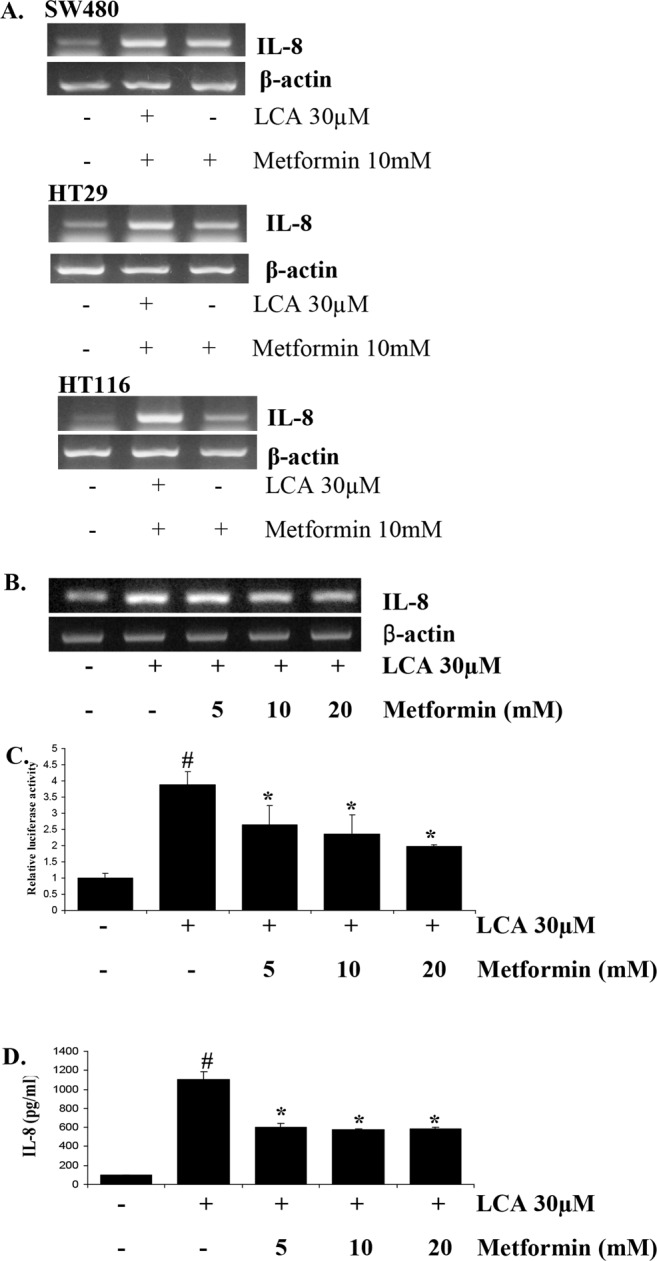
Metformin inhibits LCA-induced IL-8 expression in HCT116 CRC cells. (**A**) Three CRC cell lines, SW480, HT29, HCT116, were treated with 10 mM metformin for 1 h. The cells were then incubated with 30 µM LCA for 4 h. Then, mRNA was extracted, and IL-8 expression was evaluated by RT-PCR. (**B**) HCT116 cells pretreated with 0–20 mM metformin for 1 h were exposed to 30 µM LCA for 4 h. The cells were then extracted for mRNA, and IL-8 expression was evaluated by RT-PCR. (**C**) HCT116 cells transfected with the pGL2-IL-8 plasmid were pretreated with 0–20 mM metformin for 1 h. The cells were then incubated with 30 µM LCA for 12 h, lysed with passive lysis buffer, and submitted for the luciferase assay. (**D**) HCT116 cells were pretreated with 0–20 mM metformin for 1 h and incubated with 30 µM LCA for 24 h. The culture media were harvested and utilized for ELISA to check the levels of secreted IL-8 cytokine. Agarose gel images were cropped for clarity of the presentation. ^#^*P* < 0.05 versus control; *P < 0.05 versus LCA. The above data represent the means ± SD from triplicate measurements.

To investigate the inhibitory effect of metformin on IL-8 upregulation, three different CRC cell lines, HCT116, SW480, and HT29, were pretreated with 10 mM metformin and then treated with 30 µM LCA for 4 h. Total mRNA was then extracted from the cells, and IL-8 expression was evaluated by RT-PCR. As shown in Fig. 1A, metformin inhibited LCA-induced IL-8 expression in all three CRC cell lines. However, we selected the HCT116 cell line for further studies because the inhibitory effect of metformin was the strongest in this cell line and we had previously studied the molecular mechanism of LCA-induced IL-8 upregulation in this cell line as described in the previous study^28^.

HCT116 cells pretreated with metformin at 0–20 mM were incubated with 30 µM LCA, and then RT-PCR and luciferase analyses were performed to detect IL-8 transcriptional levels. As shown in Fig. 1B, IL-8 upregulation induced by LCA treatment was significantly inhibited by pretreatment with metformin in a dose-dependent manner. We confirmed this inhibitory effect of metformin by the IL-8 promoter luciferase assay; we found that metformin abrogated the promoter activity stimulated by LCA in a dose-dependent manner (Fig. 1C). Moreover, using a human-specific IL-8 ELISA, we demonstrated that metformin prevented HCT116 cells from producing and secreting IL-8 under LCA treatment (Fig. 1D).

### MAPK signaling is not involved in metformin’s inhibition of LCA-induced IL-8 upregulation in HCT116 CRC cells

**Figure 2:**
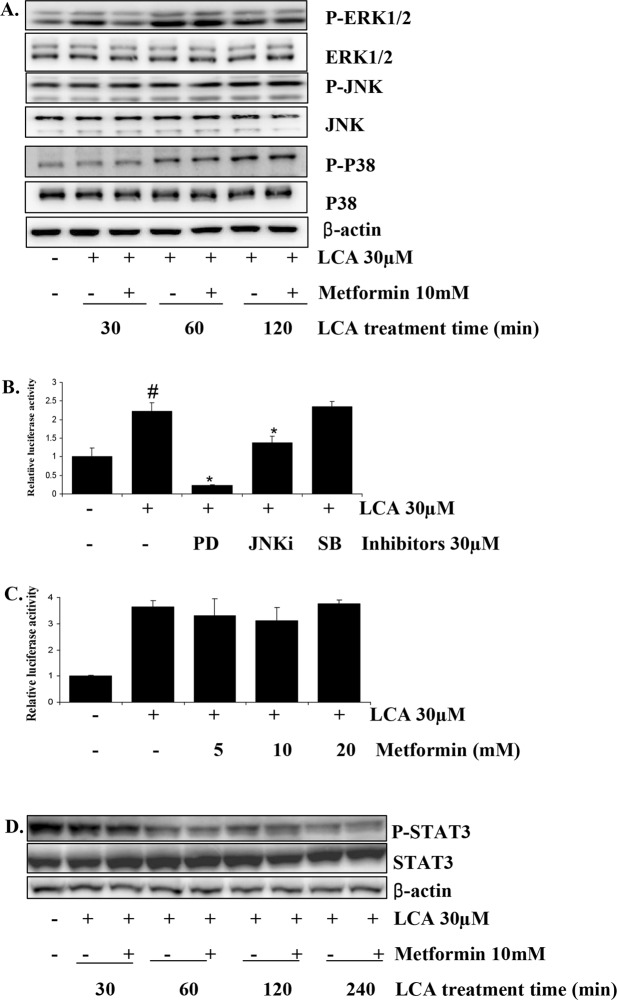
Metformin inhibition of LCA-induced IL-8 expression in HCT116 CRC cells is not mediated through MAPK signaling. (**A**) HCT116 cells pretreated with 10 mM metformin for 1 h were exposed to 30 µM LCA for 30 min, 60 min, or 120 min. The cells were then extracted for protein and tested for the levels of phosphorylated Erk1/2, JNK, and P-38. (**B**) HCT116 cells transfected with the pGL3-AP1 promoter and pRLTK plasmids were pretreated with 30 µM of PD, JNKi, or SB for 1 h. The cells were then incubated with 30 µM LCA for 12 h and lysed with passive lysis buffer to check AP-1 activity by dual luciferase assay. (**C**) HCT116 cells transfected with the pGL3-AP1 promoter and pRLTK plasmids were pretreated with 0–20 mM metformin for 1 h. The cells were then incubated with 30 µM LCA for 12 h and checked for AP-1 activity by dual luciferase assay. (**D**) HCT116 cells were pretreated with 10 mM metformin for 1 h and incubated with 30 µM LCA for 0–240 min. The cells were then extracted for protein and checked for total and phosphorylated STAT3 protein. Blot images were cropped for clarity of the presentation. ^#^*P* < 0.05 versus control; *P < 0.05 versus LCA. The above data represent the means ± SD from triplicate measurements.

In our previous study, we demonstrated that Erk1/2 is the main signal mediating the LCA-induced IL-8 upregulation in the HCT116 cell line^28^. The result of an AP-1 promoter assay using chemical inhibitors (SB, PD, JNKi) that specifically block LCA-activated P38, ERK1/2, JNK signaling, respectively (Supplementary Fig. 3), showed that Erk1/2 activation primarily stimulates AP-1, one of the essential transcription factors for regulating IL-8 expression (Fig. 2B). Thus, we hypothesized that metformin could affect the Erk1/2 signaling that is activated by LCA, preventing AP-1 activation to inhibit the IL-8 expression stimulated by LCA. However, the result in Fig. 2A shows that metformin only inhibited Erk1/2 activation during the first 30 min of LCA treatment, not at later time points. Metformin also did not affect either JNK or P38 MAPK signaling stimulated by LCA (Fig. 2A and Supplementary Fig. 1). AP-1 activity stimulated by LCA was not influenced by metformin pretreatment at 5–20 mM (Fig. 2C). These results indicate that metformin inhibition of LCA-induced IL-8 upregulation in HCT116 CRC cells was not mediated by Erk1/2/AP-1 signaling.

We continued to test metformin’s influence on the STAT3 signaling activation that we found to be a result of Erk1/2 stimulation under LCA treatment in our previous study. The results showed that metformin could not rescue the STAT3 activation that was inhibited by LCA treatment, as we previously described^28^ (Fig. 2D and Supplementary Fig. 1). This finding means that neither Erk1/2/AP-1 nor Erk1/2/STAT3 signaling is involved in the mechanism of metformin inhibition of LCA-induced IL-8 upregulation in HCT116 CRC cells.

### Metformin inhibits NF-κB activity that is involved in LCA-induced IL-8 upregulation in HCT116 CRC cells

**Figure 3:**
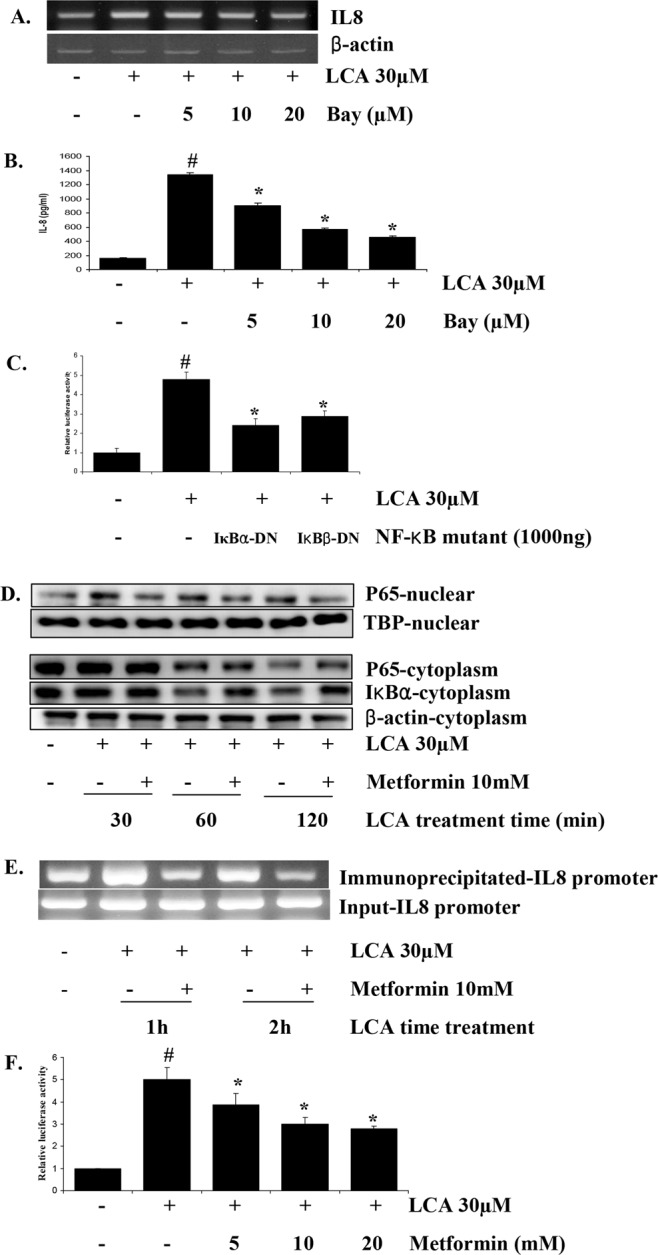
Metformin inhibits LCA-induced IL-8 expression in HCT116 CRC cells via NF-κB signaling. (**A**) HCT116 cells were treated with 0–20 µM Bay and incubated with 30 µM LCA for 4 h. The cells were then extracted for mRNA, and IL-8 expression was checked by RT-PCR. (**B**) HCT116 cells were treated with 0–20 µM Bay for 1 h and then incubated with 30 µM LCA. After 24 h, cultured media were harvested and evaluated specifically for human IL-8 by ELISA. (**C**) Dominant mutants of IκBα or IκBβ were cotransfected with the pGL2-IL-8 plasmid and pRLTK plasmid into HCT116 cells. The cells were then incubated with LCA for 12 h, and the IL-8 promoter activity was evaluated by dual-luciferase assay. (**D**) HCT116 cells were pretreated with metformin for 1 h, and then 30 µM LCA was added for 30, 60, or 120 min. The cells were then harvested, and nuclear and cytoplasmic proteins were fractionated to check for P65 and Ikβα by Western blot analysis. (**E**) A 200 bp fragment of the IL-8 promoter immunoprecipitated by an NF-κB P65 specific antibody and the DNA input of CHIP assay were quantitated via PCR. (**E**) HCT116 cells transfected with the pGL3-NF-κB and pRLTK plasmids were treated with 0–20 mM metformin for 1 h. Subsequently, 30 µM LCA was added to the cells for 12 h, and NF-κB promoter activity was tested by dual luciferase assay. Agarose gel and blot images were cropped for clarity of the presentation. ^#^*P* < 0.05 versus control; *P < 0.05 versus LCA. The above data represent the means ± SD from triplicate measurements.

The IL-8 promoter region contains binding sites for the transcription factors NF-κB, AP-1, and CCAAT/enhancer-binding protein. Abundant research has demonstrated that NF-κB and AP-1 are the most essential factors involved in IL-8 expression regulation in human melanoma cell lines and tumors^29–32^. Thus, we checked the involvement of NF-κB in LCA-induced IL-8 upregulation in HCT116 cells. The results in Fig 3A,B show that a specific inhibitor of NF-κB signaling, Bay11-7082, strongly abrogated IL-8 upregulation stimulated by LCA at both the transcription and the protein levels. Additionally, results from an IL-8 promoter assay showed that dominant mutants of IκBα (IκBα-DN) and IκBβ (IκBβ-DN), two factors involved in NF-κB signaling, could block the activation of the IL-8 promoter that is induced by LCA (Fig. 3C). These data confirmed that IL-8 expression upregulated by LCA treatment was mediated by NF-κB signaling. Therefore, we hypothesized that NF-κB could be a target of metformin in obstructing the LCA stimulatory effect on IL-8 expression.

To track NF-κB activation, we fractionated nuclear and cytoplasm proteins and checked the protein levels of NF-κB P65 and IκBα in both fractions. The results of β-actin and TBP in both fractions showed that the nuclear and cytoplasm proteins were well fractionated (Supplementary Fig. 5). Therefore, the results indicated that metformin prevented NF-κB P65 from translocating from the cytoplasm to the nucleus (Fig. 3D, and Supplementary Fig. 2). Whereas LCA augmented IκBα degradation to release P65 to the nucleus to form activated NF-κB, metformin delayed this process (Fig. 3D, and Supplementary Fig. 2). Additionally, our chromatin immunoprecipitation (CHIP) assay results also confirmed that LCA encouraged the interaction of the NF-κB transcription factor with the IL-8 promoter for upregulation of IL-8 gene expression, but metformin strongly inhibited this interaction (Fig. 3E), These data clearly indicated that metformin suppressed the activation of NF-κB signaling that is stimulated by LCA treatment.

Additionally, an NF-κB promoter assay determined that metformin reduced the NF-κB promoter activity stimulated by LCA treatment in a dose-dependent manner (Fig. 3F). These results mean that metformin inhibited LCA-induced IL-8 upregulation in HCT116 CRC cells through suppressing NF-κB activity.

### Metformin inhibits ROS production induced by LCA, in turn inhibiting NF-κB activity to abrogate the IL-8 upregulation stimulated by LCA

**Figure 4:**
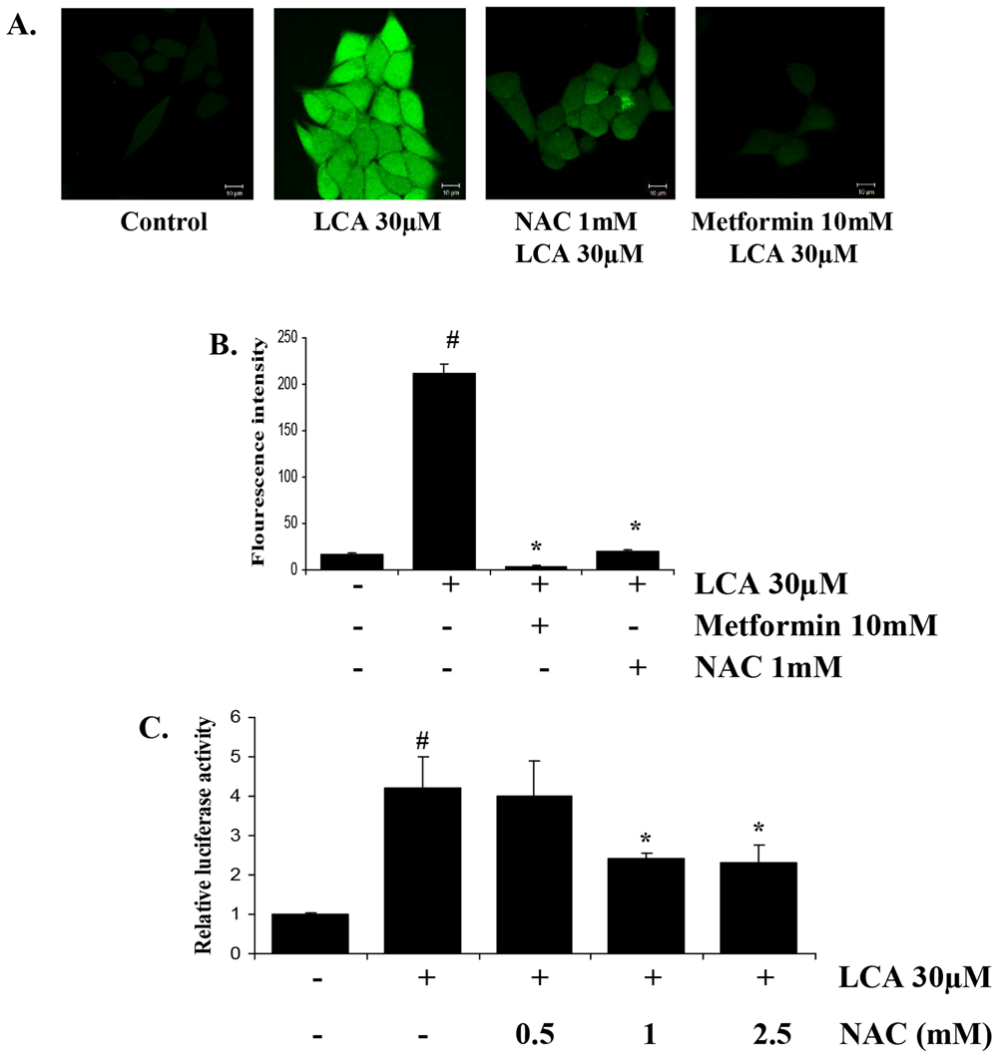
Metformin blocks ROS production induced by LCA, in turn inhibiting NF-κB activity to diminish LCA-induced IL-8 expression in HCT116 CRC cells. (**A**, **B**) Representative images (80x) and statistically quantitative values of ROS production by confocal microscope. Scale bar, 10 µM. (**C**) pGL3-NF-κB plasmid-transfected HCT116 cells were treated with 0–2.5 mM NAC for 1 h. Subsequently, 30 µM LCA was added for 12 h, and the cells were harvested to evaluate the NF-κB promoter activity by dual luciferase assay. ^#^*P* < 0.05 versus control; *P < 0.05 versus LCA. The above data represent the means ± SD from triplicate measurements.

Because ROS are well-known as a stimulator of the transcription factor NF-κB^33^, we checked the effects of LCA and metformin on ROS production. We found that LCA strongly stimulated ROS production and that both metformin and NAC pretreatment completely negated the ROS stimulated by LCA (Fig. 4A,B). Moreover, an NF-κB promoter assay showed that treatment with the ROS scavenger NAC at 1–2.5 mM abrogated LCA-induced NF-κB activation (Fig. 4C). NAC or an NADPH oxidase inhibitor (DPI) did not cause any effect on ERK1/2 or STAT3 signaling activated by LCA (Supplementary Fig. 4). These results mean that metformin blocks ROS production, which in turn obstructs NF-κB activation, for further IL-8 expression stimulated by LCA.

### Metformin inhibits ROS production stimulated by LCA by suppressing NADPH oxidase activity

**Figure 5:**
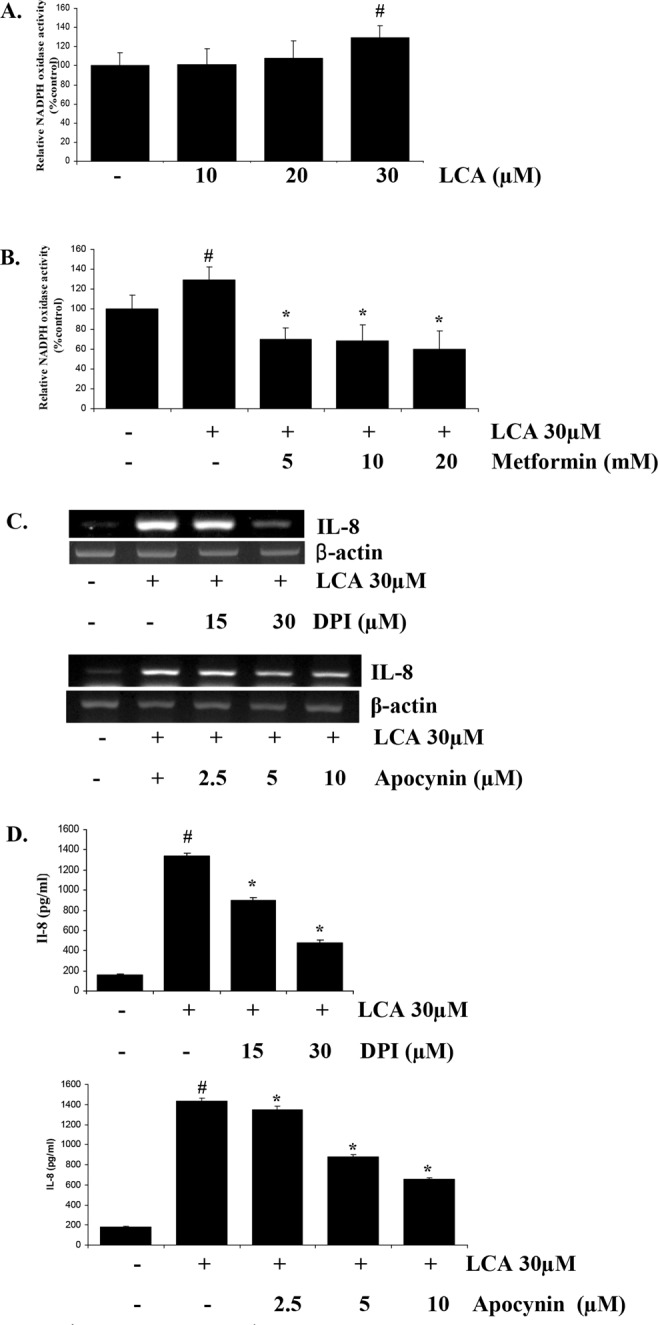
Metformin suppresses NADPH oxidase, in turn blocking LCA-stimulated ROS production. (**A**) HCT116 cells were treated with 0–30 µM LCA for 4 h and then lysed with NADPH buffer to extract protein for the NADPH oxidase assay. (**B**) HCT116 cells were pretreated with 0–20 mM metformin for 1 h, and then 30 µM LCA was added for 4 h. The cells were then lysed with NADPH buffer, and NADPH oxidase activity was checked. (**C**) HCT116 cells were treated with specific NADPH oxidase inhibitors, DPI and apocynin, for 1 h. The cells were then treated with 30 µM LCA for 4 h and checked for IL-8 expression by RT-PCR. (**D**) HCT116 cells pretreated with 15–30 µM DPI and 2.5–10 µM apocynin for 1 h were incubated with 30 µM LCA for 24 h. The cultured media were then harvested and used for secreted IL-8 quantification by ELISA. Agarose gel images were cropped for clarity of the presentation. ^#^*P* < 0.05 versus control; *P < 0.05 versus LCA. The above data represent the means ± SD from triplicate measurements.

NADPH oxidase is one of the robust sources of ROS production, and thus, we hypothesized that metformin’s blocking effect on ROS production could be derived from its effect on NADPH oxidase; we thus checked the influence of LCA and metformin on the activity of this enzyme in HCT116 CRC cells. As we expected, an NADPH oxidase assay showed that treatment with 30 µM LCA significantly stimulated NADPH oxidase activity (Fig. 5A), but this stimulation was strongly abrogated by metformin pretreatment (Fig. 5B). Moreover, specific inhibitors of NADPH oxidase, DPI and apocynin, significantly reversed LCA’s effect on IL-8 expression (Fig. 5C,D). These results indicate that metformin suppresses NADPH oxidase activity, decreasing ROS production, blocking NF-κB activation, and, finally, obstructing LCA’s stimulatory effect on IL-8 expression.

### Metformin inhibits the LCA-induced angiogenesis activity in HCT116 CRC cells

**Figure 6:**
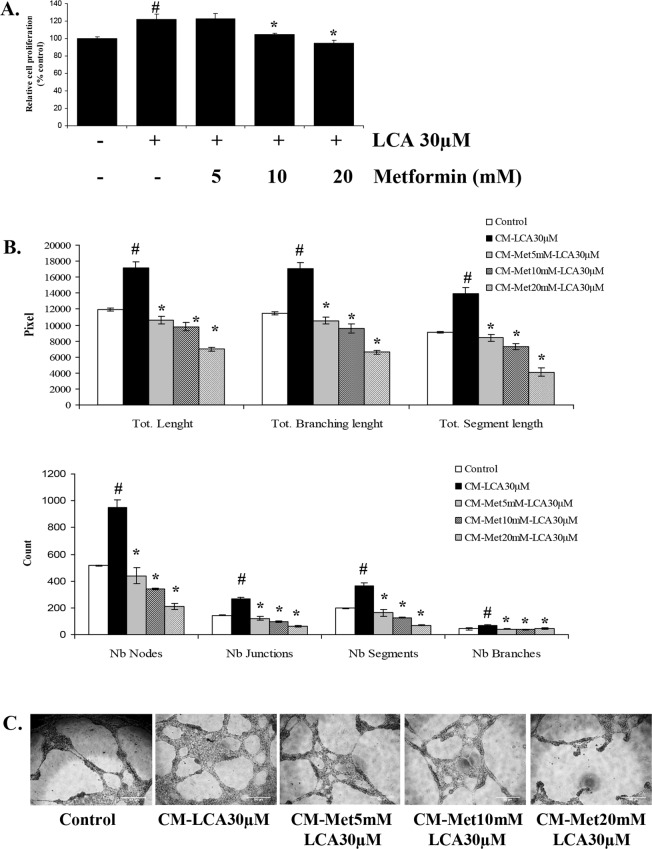
Metformin inhibits proliferation and tubelike formation of ECV304 endothelial cells stimulated by CM derived from LCA-treated HCT116 CRC cells. (**A**) ECV304 cells were incubated in DMEM media supplemented with 10% FBS for 24 h. Then, CM derived from LCA-treated HCT116 cells with and without 5–20 mM metformin (Met) pretreatment was added for another 24 h. Cell proliferation was checked by cell viability, proliferation & cytotoxicity assays. (**B**, **C**) Quantitative data and representative images (10x) of the tube formation assay. ECV304 cells were seeded on Matrigel-coated wells of a 96-well plate and then incubated with CM derived from LCA-treated HCT116 cells with and without 5–20 mM metformin pretreatment. After 12 h, the cells were observed with a Nokia microscope to check the formation of tubelike structures. Scale bar, 500 µM. ^#^*P* < 0.05 versus control; *P < 0.05 versus LCA. The above data represent the means ± SD from triplicate measurements.

In our previous study, we established that IL-8 production stimulated by LCA stimulates angiogenesis in tumor environments by stimulating endothelial cell proliferation as well as tubelike formations^28^, and in this study, we saw that metformin inhibited LCA-induced IL-8 expression. Therefore, to assess metformin’s effect on CRC progression, we compared the influence of CM derived from LCA-treated HCT116 cells (CM-LCA) with CM from LCA-treated cells with metformin pretreatment (CM-Met-LCA) on the proliferation and formation of capillary-like tubes in ECV304 endothelial cells. As shown in Fig. 6A, the proliferation rates of ECV304 cells incubated with CM-Met-10mM-LCA and CM-Met-20mM-LCA were significantly slower than the rates for cells grown in CM-LCA. Similarly, tubelike formation of endothelial cells enhanced by CM-LCA was also impeded in cells treated with CM-Met-LCA in a metformin dose-dependent manner (Fig. 6B,C, and Supplementary Fig. 8). These results demonstrated that metformin inhibits LCA-induced IL-8 expression in HCT116 CRC cells, in turn obstructing LCA’s stimulatory effect on endothelial cell proliferation and tubelike formation in tumor environments.

## Discussion

Metformin has attracted much researcher interest because of its renewed application in cancer defense. There is substantial epidemiological evidence of its benefits in cancer treatment from both preclinical and clinical studies. However, metformin’s action mechanism in cancer progression remains an active area of research. In the present study, we describe for the first time the novel mechanism of metformin acting on HCT116 CRC cells under LCA presence, which is a proven strong tumor promoter that triggers CRC development. This study supplies strong evidence for metformin as a promising drug for cancer treatment.

IL-8 overexpression has been identified as one of the prospective markers in CRC screening. Our previous study demonstrated that LCA strongly stimulated IL-8 expression in CRC cells, in turn increasing the angiogenesis of cancer cells by augmenting endothelial cell proliferation and tubelike formation^28^; here, we revealed that metformin could block this stimulatory effect of LCA on CRC cells. In several previous studies, metformin was demonstrated to inhibit the inflammatory response in both normal and cancer cells. Metformin inhibited the IL-1β-induced release of the proinflammatory cytokines IL-6 and IL-8 in vascular wall cells^34^ and endometriotic stromal cells^35^, explaining in part metformin’s benefits in cardiovascular and endometriosis disease treatment. In esophageal cancer cells or cancer stem cells, metformin reversed the expression of several inflammatory genes, including IL-8, Lin28B, Let-7, IL-1α, IL-1β, IL-1, and vascular endothelial growth factor (VEGF), preventing cellular proliferation and transformation^36,37^. Combined with the present results that metformin inhibited LCA-induced IL-8 upregulation, we find that inhibiting the inflammatory response is one of the mechanisms for how metformin delays cancer progression.

The NF-κB/Rel transcription factor has long been demonstrated to have an essential role in the cellular inflammatory response in general^38^ and IL-8 expression specifically, and our findings demonstrated that LCA-induced IL-8 upregulation in HCT116 CRC cells was mediated by the NF-κB pathway and that metformin blocked this pathway activation to obstruct IL-8 overexpression. There have been abundant reports demonstrating that metformin diminishes NF-κB, inhibiting inflammatory responses^34^, cancer stem cell proliferation and transformation^37^, and the expression of adhesion molecular genes such as VCAM-1, ICAM-1, E-Selectin, and MCP-1 in endothelial cells^39^. Hattori *et al*. described the inhibitory mechanism of metformin on TNFα-induced NF-κB by which metformin, through AMPK activation, attenuated the phosphorylation and subsequent degradation of IκBα by inhibiting IκB kinase activity, suppressing cytokine-induced NF-κB nucleus translocation^39^. Our results also show the same mechanism of metformin inhibiting LCA-induced NF-κB in HCT116 CRC cells with the delay of IκBα degradation and NF-κB/P65 nucleus translocation to enhance IL-8 expression (Fig. 3).

There is prior evidence that metformin’s primary effect is in mitochondria, where it interrupts the respiratory electron chain and reduces ATP production, activating AMP kinase^15^. Because the respiratory electron chain in mitochondria is an important source of ROS, many studies demonstrated that metformin attenuates the elevated ROS production related to DNA damage and mutations^15^, inflammatory signaling^40^, and cancer progression^41^. This inhibitory effect of metformin was interpreted as inhibition of NADH ubiquinone oxidoreductase, an important enzyme in the mitochondrial respiratory chain,^40^ and protein kinase C^42^ or enhancement of serial antioxidant enzymes such as catalase, superoxide dismutase, and glutathione peroxidase^43,44^. Buldak *et al*. revealed that the reduction effect of metformin on ROS production is derived from its inhibition of NADPH oxidase, a key producer of ROS^42^. Here, our results also demonstrated that metformin completely blocks ROS production by deactivating NADPH oxidase stimulated by LCA treatment (Fig. 5). When ROS production is not elevated, the activation of NF-κB signaling also disappears, ultimately preventing the IL-8 upregulation induced by LCA in HCT116 CRC cells.

IL-8 is one of the important stimulators of angiogenesis activity. This study’s findings indicate that metformin diminishes the IL-8 overexpression induced by LCA, reducing the angiogenesis exhibited via endothelial cell proliferation and tubelike formation. Even the endothelial cell model adapted in this manuscript is not representative for tumor angiogenesis *in vivo*; many studies have described the anti-angiogenic activity of metformin in tumor models with the involvement of abundant numbers of angiogenesis-associated proteins such as VEGF^45,46^, cyclooxygenase 2, CXC chemokine receptor 4, IL-8, angiogenin, and TIMP-1^47^. Here, not only does metformin annul tubelike formation stimulated by LCA-induced IL-8 overexpression, it also strongly reduces the formation of capillary-like tubes compared with control CM. Our result with CM derived from metformin-treated HCT116 cells also confirmed that these CMs strongly inhibited tubelike formation of ECV304 endothelial cells in a dose-dependent manner (Supplementary Fig. 6). These results drive us toe hypothesize that IL-8 inhibition should not be the unique target of metformin in endothelial cells in this model. Thus, investigation of affected molecules other than IL-8 in relation to the anti-angiogenesis activity of metformin in CRC cells could be very promising.

In conclusion, this study demonstrates that metformin inhibits LCA-induced IL-8 upregulation by suppressing NADPH oxidase, an important enzyme in ROS production, and NF-κB activation in HCT116 CRC cells and diminishes endothelial cell proliferation and tubelike formation in tumor microenvironments. These results augment our understanding of the defense mechanism of metformin against cancer progression and contribute to developing metformin as a new strategy for CRC treatment.

## Materials and Methods

### Cell culture conditions and materials

HCT116 human colon carcinoma cells were obtained from the American Type Culture Collection (Rockville, MD, USA). The cells were cultured at 37 °C in a 5% CO_2_ atmosphere in McCoy’s 5 A medium supplemented with 10% fetal bovine serum (FBS) and 1% penicillin-streptomycin. LCA was obtained from Sigma Chemical Co. (St. Louis, MO, USA) and dissolved in dimethyl sulfoxide (DMSO) as 30 mM stock solutions. Metformin was purchased from Santa Cruz Biotechnology (Dallas, TX, USA). Bay11-7082 (Bay), diphenyleneiodonium (DPI), PD98059 (PD), JNKi, and SB203580 (SB) from Calbiochem (San Diego, CA, USA) and apocynin from Selleckchem (Houston, TX, USA) were dissolved in DMSO and stored at −80 °C. N-acetyl-L-cysteine (NAC) was purchased from Sigma and was freshly prepared by dissolving in water immediately before use.

### Reverse-transcription polymerase chain reaction (RT-PCR)

RT-PCR was performed as described in a previous study^28^. The sequences of the specific primers applied for detecting IL-8 and β-actin expression were as follows: β-actin forward, 5′-AAG CAG GAG TAT GAC GAG TC-3′ and β-actin reverse, 5′-GCC TTC ATA CAT CTC AAG TT-3′ (561 bp); IL-8 forward, 5′-CAT ACT CCA AAC CTT TCC AC −3′ and IL-8 reverse, 5′-ACT TCT CCA CAA CCC TCG C −3′ (159 bp).

### Measuring IL-8 secretion

HCT116 cells (2 × 10^5^ cells/well) were seeded in a 24-well plate in McCoy’s 5 A medium containing 10% FBS at 37 °C. After 24 h, the cells were switched into new medium containing 1% FBS overnight. Metformin (5–20 mM) was added to the cells 1 h prior to 24 h of LCA treatment. Equal volumes of cell culture supernatants were collected, and the secreted cytokine IL-8 was quantified using an IL-8 ELISA kit (R&D Systems, Minneapolis, MN, USA). The IL-8 concentrations in the culture supernatants were determined by extrapolating their optical densities to the standard curve and are expressed as pg/ml.

### Promoter activity assays

HCT116 cells (3 × 10^4^) were seeded on a 48-well plate and grown to 60–70% confluence, and then IL-8 promoter/nuclear factor-kappa B (NF-κB) promoter/activator protein-1 (AP-1) promoter-luciferase reporter plasmids (pGL2-IL-8; pGL3-NF-κB; pGL3-AP-1) and pRLTK were cotransfected into the cells using the FuGENE transfection reagent (Boehringer Mannheim, Indianapolis, IN, USA). Cells were incubated in the transfection medium for 24 h, switched to new medium containing 1% FBS, and then treated with LCA for 4 h. Metformin’s inhibitory effects on IL-8 promoter, NF-κB promoter, and AP-1 promoter activity were determined by pretreating the cells with metformin for 1 h prior to adding LCA.

### Western blot analysis

Western blot analysis to check levels of phosphorylated p44/42 MAPK (Erk1/2), phosphorylated P38 MAPK, phosphorylated JNK, and phosphorylated STAT3 was performed as described in our previous study^28^. To track NF-κB activation, the Active Motif North America Nuclear Extract Kit (Carlsbad, CA, USA) was applied to fractionate the nuclear and cytoplasmic proteins of HCT116 cells. Then, 15–30 µg of protein from each fraction was resolved on 12% sodium dodecyl sulfate-polyacrylamide gels, transferred to Immobilon^®^ PVDF membranes, and subjected to the rabbit polyclonal anti-P65 antibody and the rabbit polyclonal anti IκBα antibody (Santa Cruz Biotechnology). As a control for the protein loading, TATA-box binding protein (TBP; for the nuclear fraction) and β-actin (for the cytoplasmic fraction) levels were assessed.

### Chromatin immunoprecipitation (CHIP) assay

HCT116 cells (5 × 10^5^) were cultured in 6 cm diameter Petri dishes in McCoy’s 5 A supplemented with 10% FBS for 24 h and then switched to 1% FBS supplemented McCoy’s 5 A media overnight. The cells were then treated with 10 mM metformin for 1 h and then 30 µM LCA for 1 h and 2 h. The cells were treated for 30 min at room temperature with 1% formaldehyde culture media, and then glycine was added to a final concentration of 0.125 M to block cross-linking. The cells were then washed in PBS, suspended in 1 ml swelling buffer (25 mM HEPES pH 7.8; 1.5 mM MgCl_2_, 10 mM KCl, 0.1% NP-40, 1 mM DTT, 0.5 mM PMSF, and protease inhibitor cocktail), incubated 30 min on ice, and centrifuged to obtain nuclei. The nuclei were suspended in 500 µl sonication buffer (50 mM HEPES pH 7.9, 140 mM NaCl, 1 mM EDTA, 1% Triton X-100, 0.1% Na-deoxycholate, 0.1% SDS, 0.5 mM PMSF, and protease inhibitor cocktail) and then subjected to sonication (5 times) for 3 second pulses at 30% amplitude by a Sonics Vibra-Cell sonicator to shear chromatin into 200–1000 bp fragments. Then, 15 µg chromatin from each sample was diluted to 500 µl in sonication buffer and then cleared with a 20 µl A/G agarose 50% gel slurry for 2 h at 4 °C before incubation on a rocking platform with 5 µl NF-κB p65-specific rabbit antibody (Cell Signaling). The remaining 15 µg of sheared chromatin from each sample was saved and stored for later PCR analysis as input extracts for the CHIP assay. Incubation occurred for 2 h at 4 °C and continued overnight after the addition of 20 µl protein A/G-agarose slurry. Thereafter, the agarose pellets were washed with low-salt, high-salt, and LiCl buffers. DNA/protein complexes were recovered from the pellets with elution buffer (50 mM Tris pH 8.0, 1 mM EDTA, 50 mM NaHCO_3_ with 1% SDS, and 0.32 sucrose) at room temperature for 30 min. Then, the CHIP elutions and input extracts were incubated 5 h at 65 °C with 0.2 M NaCl for cross-link reversion. The samples were treated with RNase A and proteinase K, extracted with phenol/chloroform, and ethanol precipitated. The pelleted DNA was washed with 70% ethanol and dissolved in 20 µl deionized water. One microliter of the obtained DNA solutions was used for the PCR reactions to quantitate immunoprecipitated promoter fragments with a primer pair that amplifies the −178 bp to + 48 bp region on the IL-8 promoter, which contains the NF-κB binding site (forward: 5′-GGT ACC GAA AAC TTT CGT CA-3′; Reverse: 5′-CTC GAG CTA GAA AGC TTG TGT-3′).

### Measuring intracellular hydrogen peroxide (H_2_O_2_)

Intracellular H_2_O_2_ production was measured using 5- and 6-carboxy-2′,7′-dichlorodihydrofluorescein diacetate (DCFDA; Molecular Probes, Eugene, OR, USA) as previously described^48^. Briefly, the cells were grown in serum-starved McCoy’s 5A medium supplemented with 1% FBS for 24 h. The cells were then switched to serum-free DMEM without phenol red and exposed to LCA for 30 min. The cells were treated with 10 mM metformin or 1 mM NAC 1 h prior to the LCA treatment to assess their effects on ROS production activated by LCA. The cells were incubated with 5 ng/ml DCFDA for 15 min and immediately observed using an LSM 510 laser-scanning confocal microscope (Carl Zeiss, Germany). The DCF fluorescence was excited at 488 nm using an argon laser, and the emission evoked was filtered with a 515 nm longpass filter.

All obtained fluorescence images taken with the LSM 510 confocal microscope were analyzed using the LSM 5 Image Browser software.

### NADPH oxidase activity assay

NADPH oxidase activity was assayed with lucigenin-enhanced chemiluminescence^49^. Briefly, HCT116 cells were harvested by cell scrapers and dounce homogenized in NADPH lysis buffer (50 mM phosphate buffer, pH 7.0, 1 mM EGTA, 150 mM sucrose, and protease inhibitors). Cell lysates were then incubated with 5 mmol lucigenin (Sigma) and 0.1 mmol NADPH (Sigma) balanced with NADPH lysis buffer. Photon emission from the chromogenic substrate lucigenin as a function of acceptance of electron/O_2_^−^ generated by the NADPH oxidase complex was measured every 2 min for 30 min in a Berthold luminometer. The enzyme activity was expressed as relative light units/µg protein in 1 min, and relative fold changes were used to indicate the activity changes.

### Angiogenesis assays

HCT116 cells were seeded at 2 × 10^5^ cells/well on a 12-well plate in McCoy’s 5A medium with 10% FBS for 24 h. The cells were switched to new medium containing 1% FBS overnight, pretreated with metformin at 5–20 mM for 1 h, and then exposed to 30 µM LCA. After a 24 h incubation, the conditioned media (CM) were collected, centrifuged, filtered, and stored at −20 °C. These CM samples were then applied to assess their effects on ECV304 endothelial cell proliferation and tubelike formation as described in our previous study^28^. Images of the tubelike formation assay were quantitatively analyzed by ImageJ software based on the definition of node, junction, branches, segment that are clearly defined in Supplementary Fig. 7.

### Statistical analysis

All results reflect a minimum of three independent experiments. We performed ANOVA for multivariable analyses and considered P < 0.05 statistically significant.

## Supplementary information

Supplementary data

Full-length gels and blots
