# Supplementary material for: Metformin inhibits lithocholic acid-induced interleukin 8 upregulation in colorectal cancer cells by suppressing ROS production and NF-kB activity
Source: Sci Rep. 2019 Feb 14;9:2003. doi: 10.1038/s41598-019-38778-2 (PMC6376015; doi:10.1038/s41598-019-38778-2)
Supplement: Supplementary file 1 — Supplementary data [file 41598_2019_38778_MOESM1_ESM.pdf]

Supplementary data and information

Metformin inhibits lithocholic acid-induced interleukin 8 upregulation in colorectal cancer cells by suppressing ROS production and NF-kB activity

Thi Thinh Nguyen[1*], Trong Thuan Ung[1*], Shinan Li[1], Sen Lian[2], Yong Xia[3], Sun Young Park[1] and Young Do Jung [1**]

[1]Department of Biochemistry, Chonnam National University Medical School, Seoyang Ro 264, Hwasun, Jeonnam 58138, Korea

[2]Department of Biochemistry and Molecular Biology, School of Basic Medical Sciences, Southern Medical University, Guangzhou, 510515, Guangdong, China

[3]Department of Urology, New York University School of Medicine, New York, NY, 10010, USA

****Corresponding author:** Young Do Jung, MD., PhD.
Department of Biochemistry, Chonnam National University Medical School, Seoyang Ro 264, Hwasun, Jeonnam 58138, Korea; Telephone: 82-61-379-2772, Fax: 82-81-379-2781, Email: ydjung@chonnam.ac.kr

* These authors contributed equally to this work

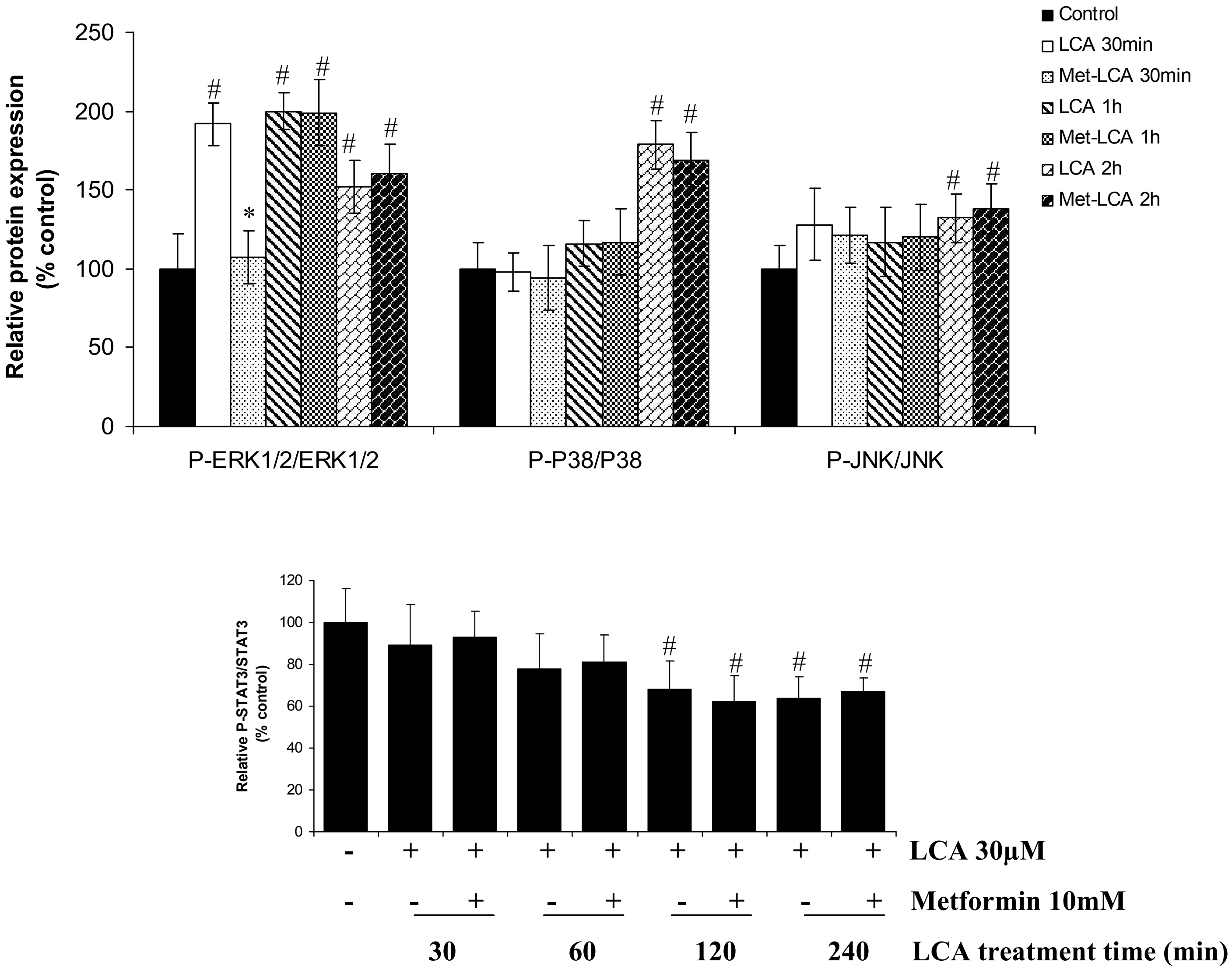

Supplementary Figure 1: Densitometric analysis to calculate the ratio of phosphorylated to total proteins, ERK1/2, JNK, P38, and STAT3. The relative levels of protein expression were normalized to β-actin used as an internal control. The above data represent the means ± SD from at least three independent experiments. #$P < 0.05$ versus control; *$P < 0.05$ versus LCA.

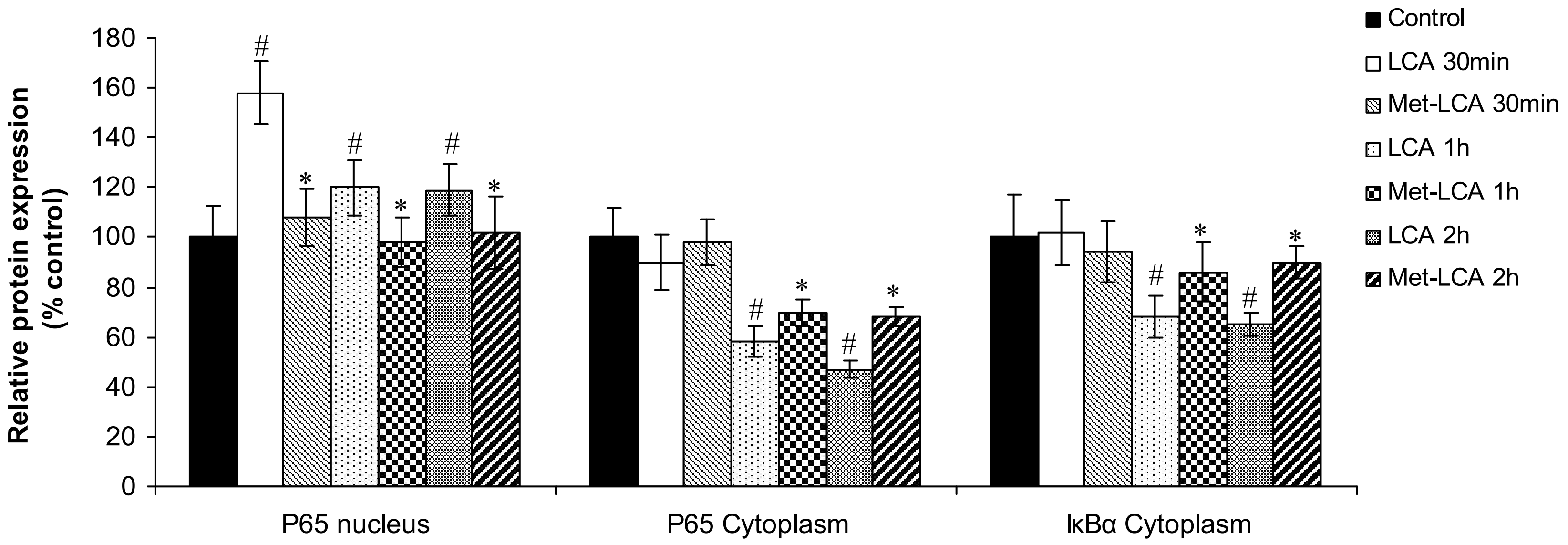

Supplementary Figure 2: Densitometric analysis to calculate the level of P65 and IκBα proteins in nucleus and cytoplasm fractions. The relative levels of P65 protein in nucleus were normalized to TBP, and cytoplasmic P65 and IκBα proteins were normalized to β-actin. The above data represent the means ± SD from at least three independent experiments. #$P < 0.05$ versus control; *$P < 0.05$ versus LCA.

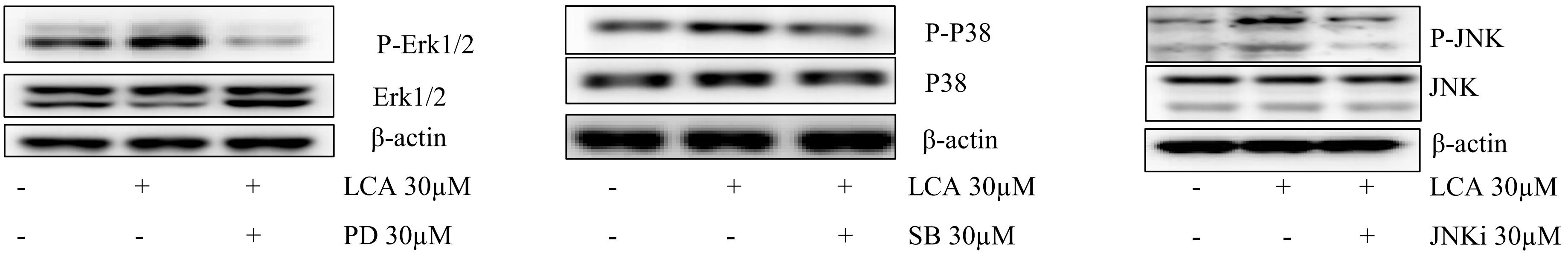

Supplementary Figure 3: Efficiency of specific chemical inhibitors of MAPK-ERK1/2 (PD), MAPK-P38 (SB), and MAPK-JNK (JNKi) signaling

HCT116 cells were pretreated with PD, SB, JNKi for 1 hour and then treated with LCA for 1h with PD-pretreated cells and 2h with SB or JNKi-pretreated cells. The cells were then harvested, extracted protein and checked the level of phosphorylated Erk1/2, P38, and JNK proteins.

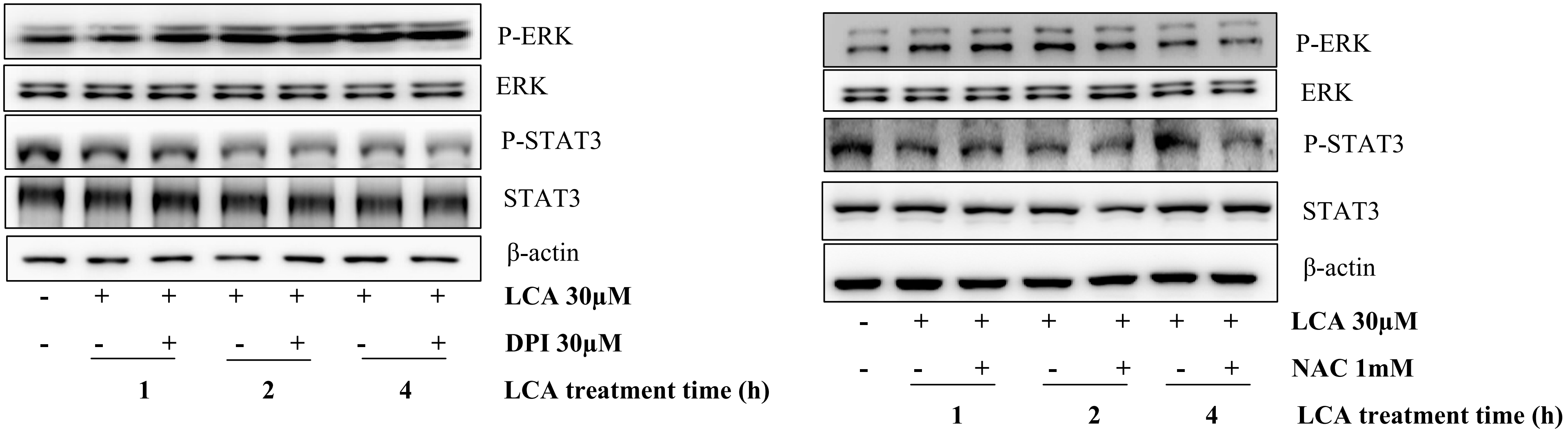

Supplementary Figure 4: HCT116 cells were pretreated with DPI 30μM or NAC 1mM for 1h. The cell were then treated with LCA for 1h, 2h and 4h, and then harvested, extracted for protein to check phosphorated protein levels of Erk1/2, and STAT3.

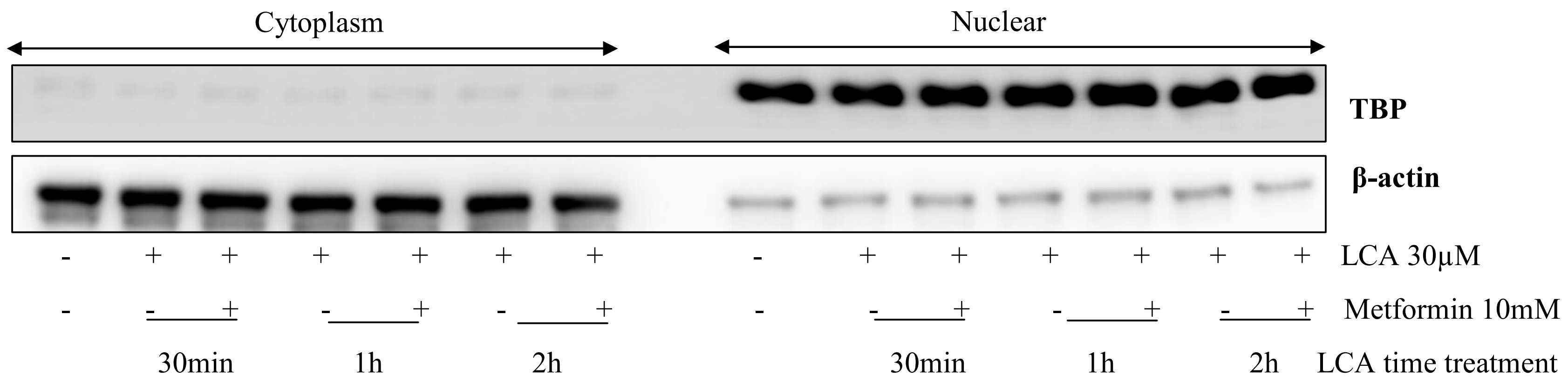

Supplementary Figure 5: TBP and β-actin protein level in cytoplasmic and nuclear fraction

HCT116 cells after harvested were fractionated to divide cytoplasmic and nuclear proteins. 10μg protein of each fraction was resolved on 12% SDS-polyacrylamide gel to check protein level of TBP and β-actin.

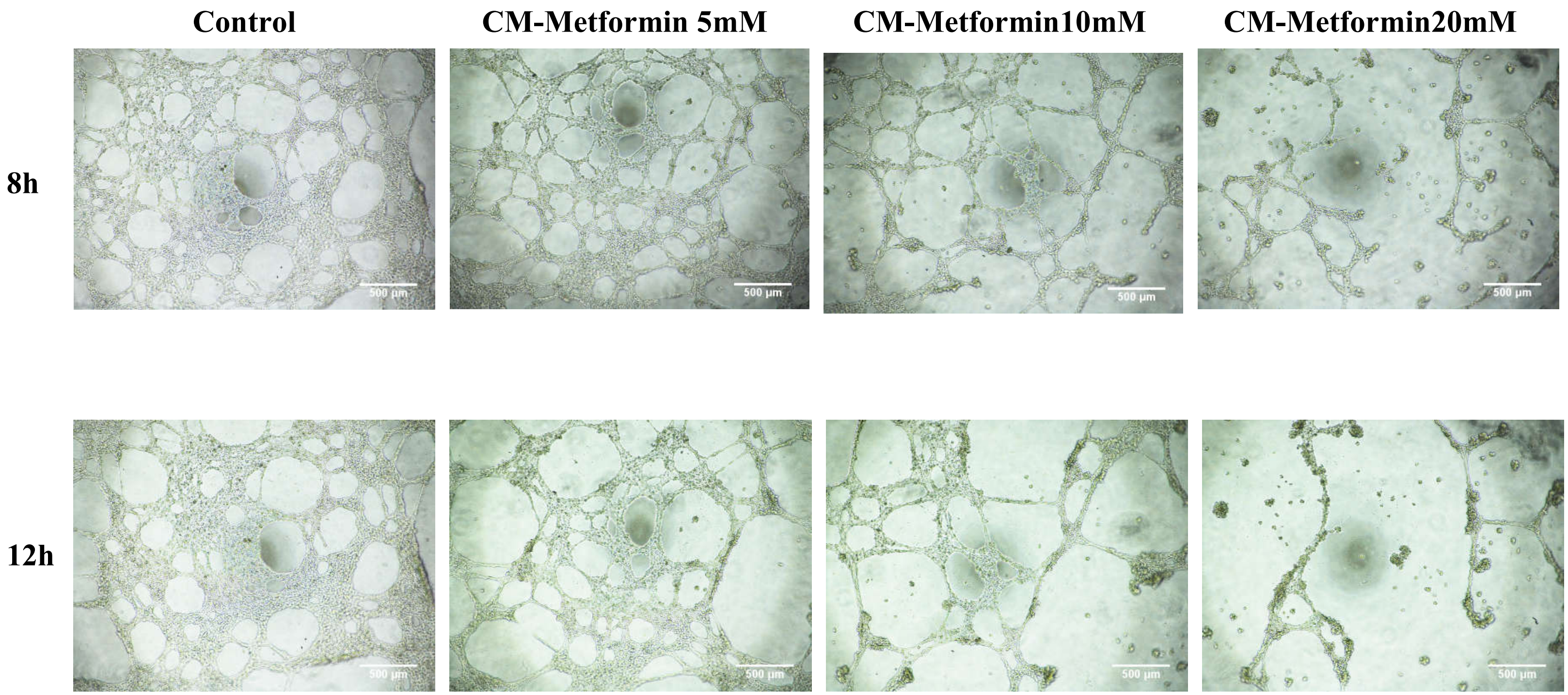

Supplementary Figure 6: CMs derived from metformin-treated HCT116 cells inhibit tube like formation of endothelial ECV304 endothelial cells

HCT116 cells were treated with metformin at 5-20mM for 24 hours and then the conditioned media was collected for tuble like formation assay. ECV304 cells were seeded on the top of matrigel pre-coated in 96 well plates, cultured for 4 hours, and then the CMs were added to the wells. The plate was incubated at 37°C and checked the formation of the capillary like tube using a Nikon microscope at 4xmagnification. Scale bar, 500μM.

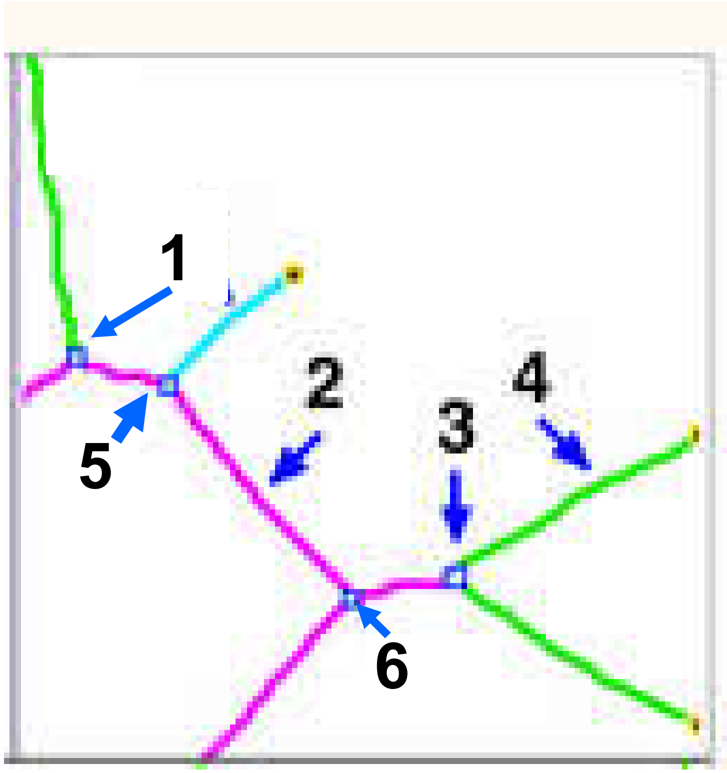

•**Nodes**, identified as pixels that had at least 3 neighbour, corresponding as bifurcation (3)

•**Junction** are points linking at least three of segments and branches (1, 3, 5, 6).

Junctions are composed by several nodes

•**Segments**: elements delimited by two junctions (2)

•**Branches**: elements delimited by a junction and one extremity (4)

Supplementary Figure 7: Analysis of tube network by ImageJ software

(http://image.bio.methods.free.fr/ImageJ/?Angiogenesis-Analyzer-for-ImageJ&lang=en&artpage=3-6#outil_sommaire_3)

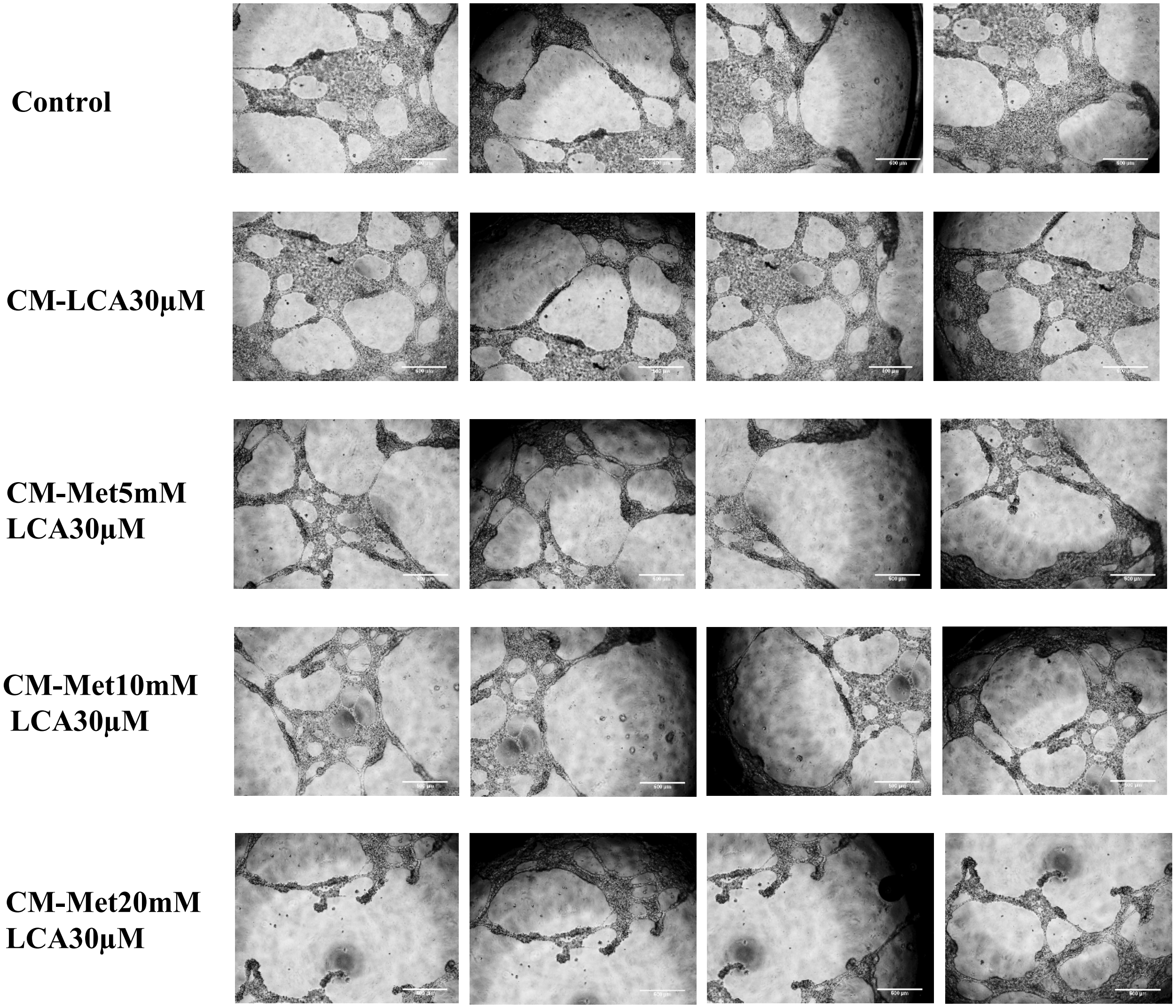

Supplementary Figure 8: Tube like formation images of ECV304 endothelial cells cultured in different conditioned media at 10x magnification. Scale bar, 500µM.

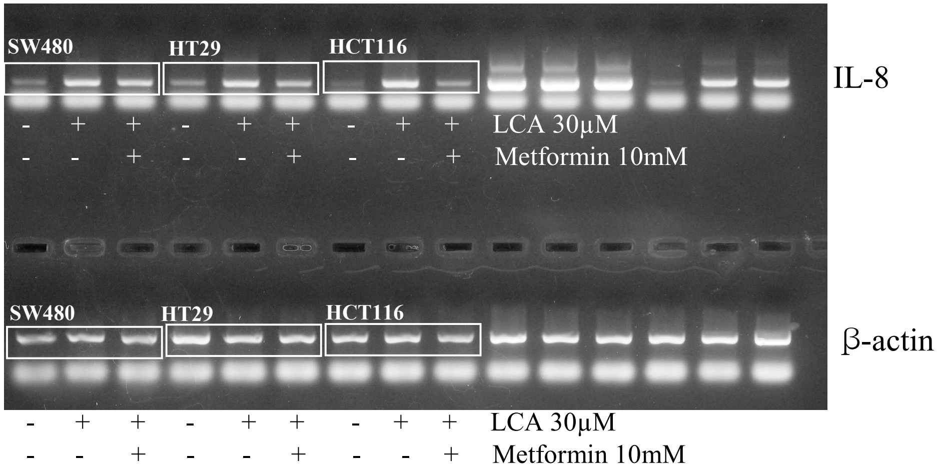

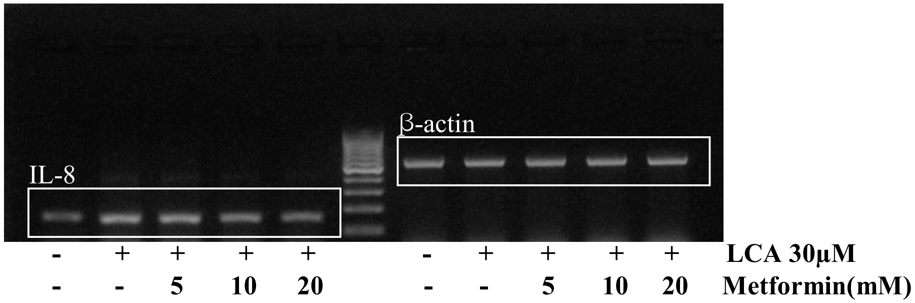

Full-length agarose gels presented in figure 1A and 1B in the article

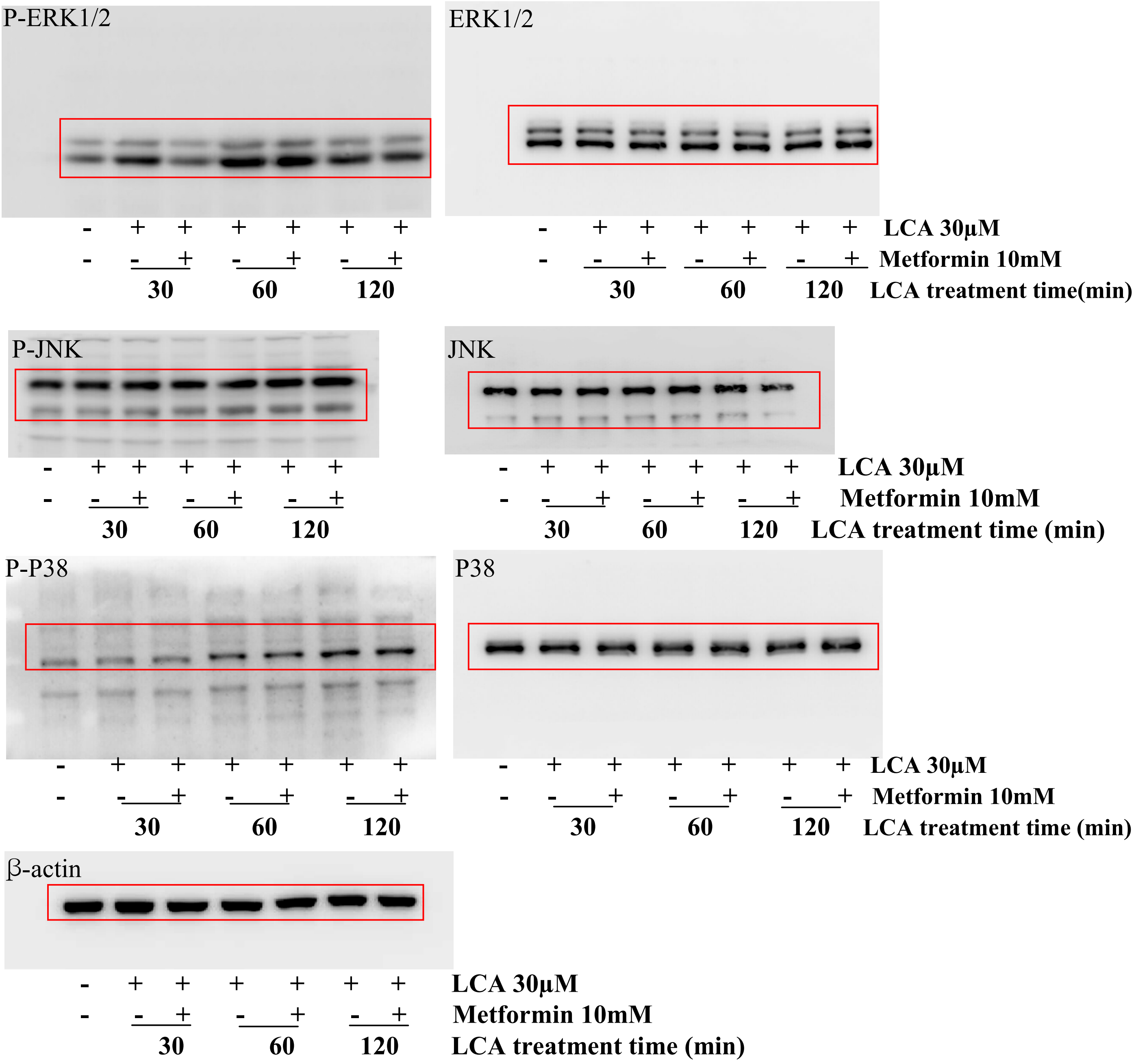

Full-length blots presented in figure 2A in the article

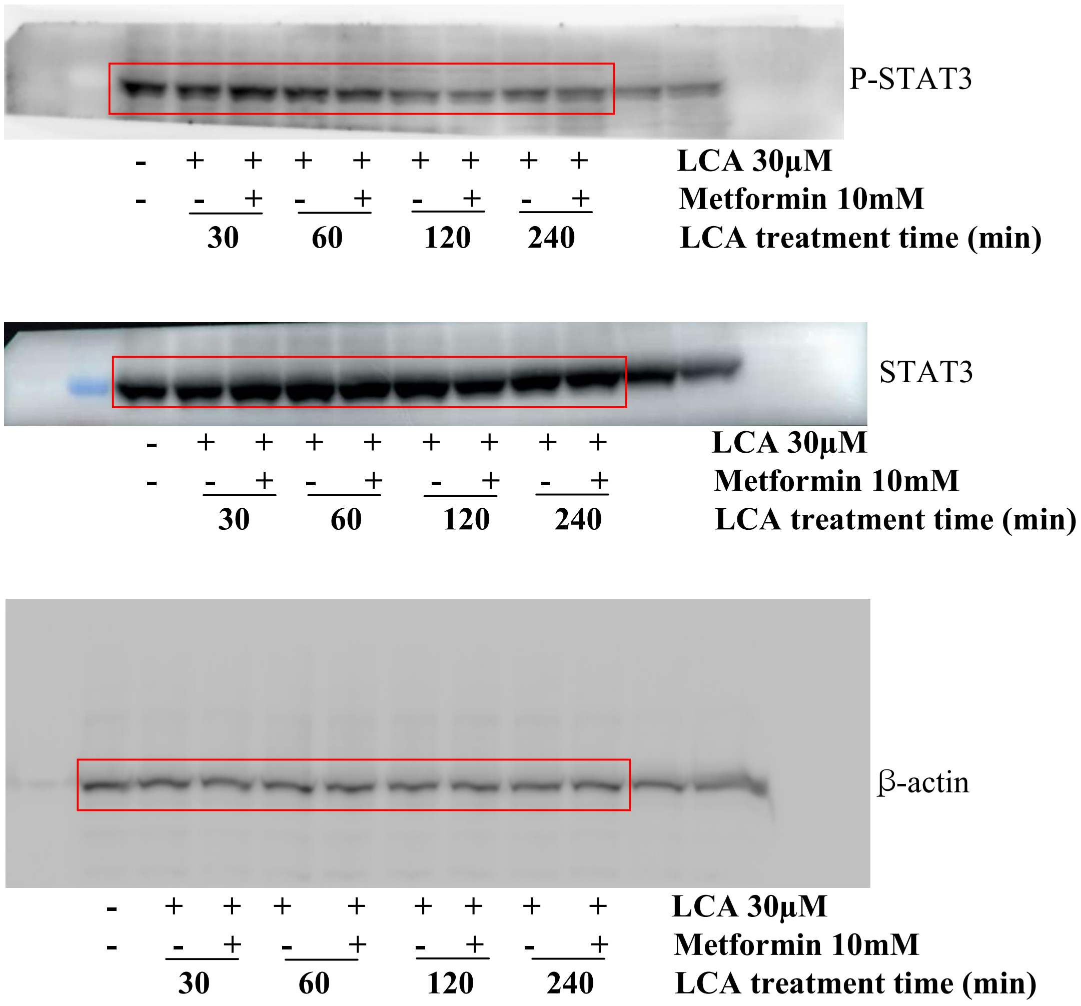

Full-length blots presented in figure 2D in the article

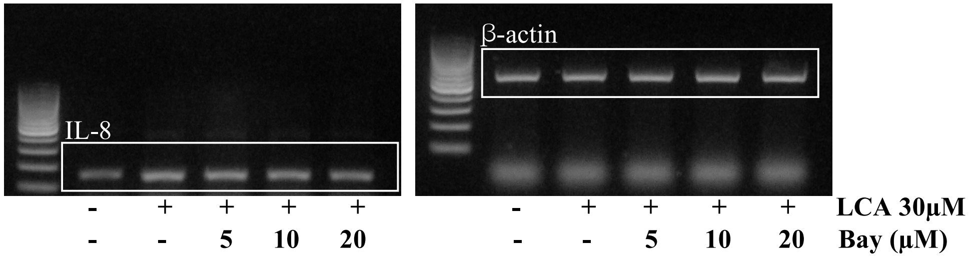

Full- length agarose gels presented in figure 3A in the article

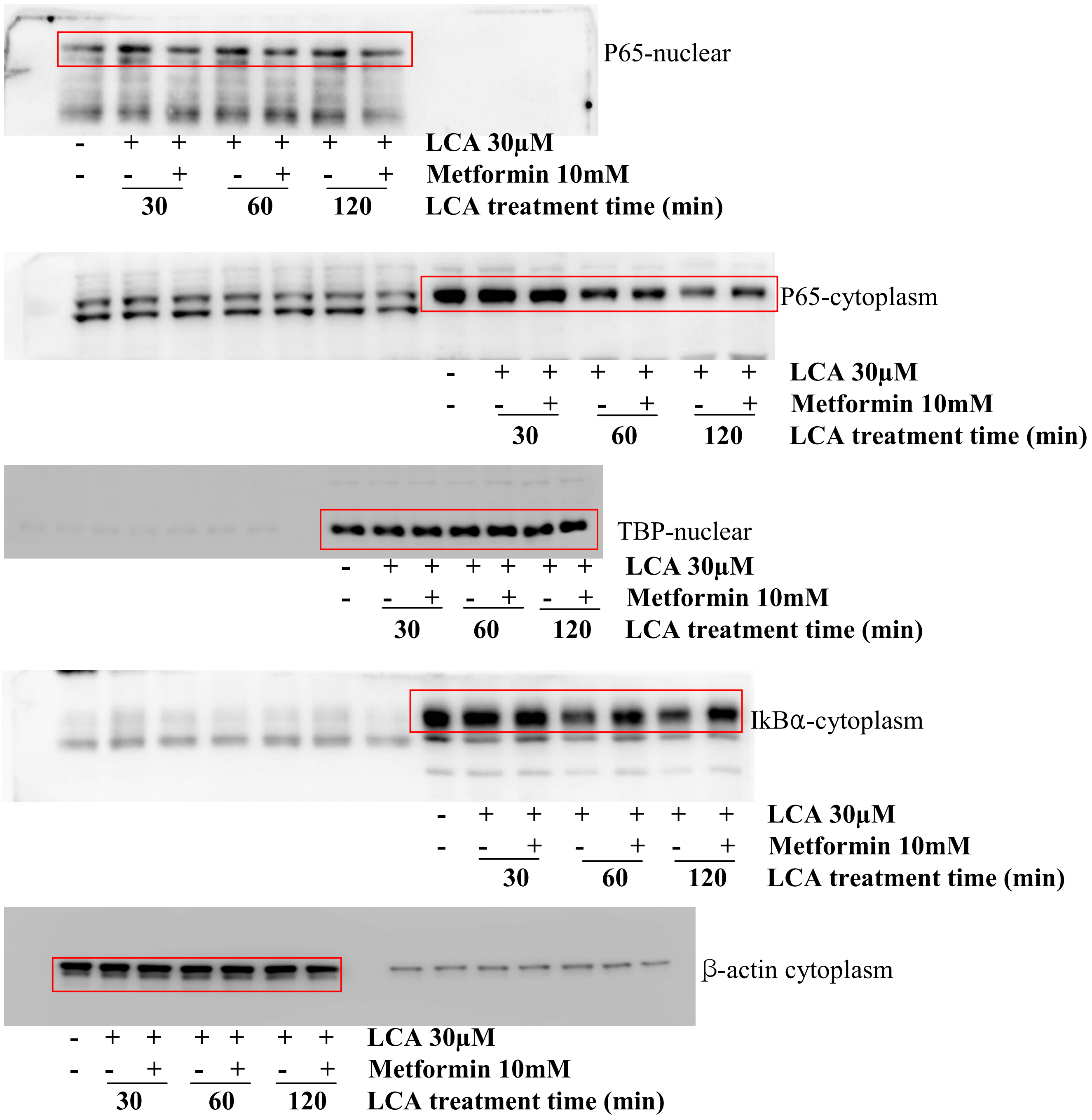

Full-length scans of the blots presented in figure 3D in the article

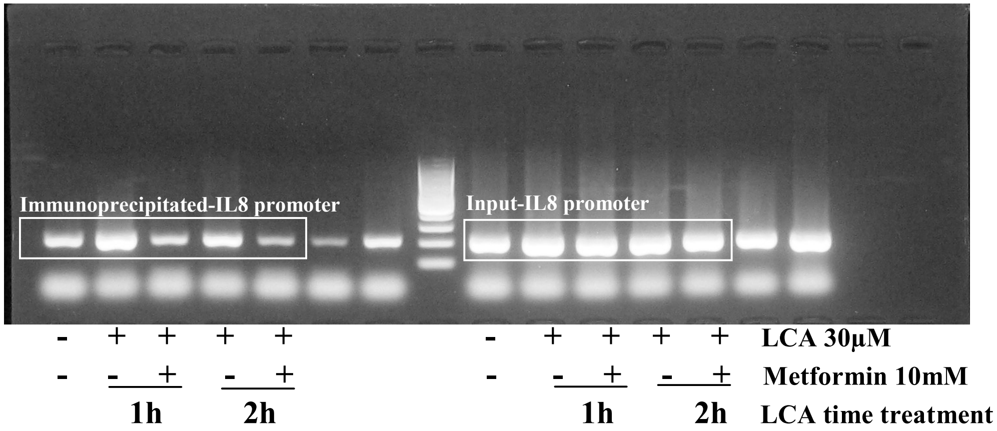

Full- length agarose gels presented in figure 3E in the article

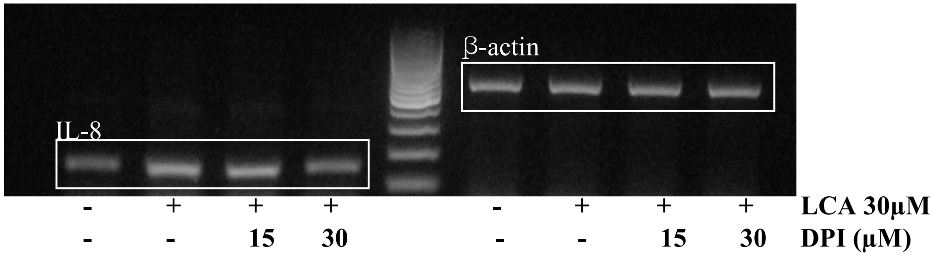

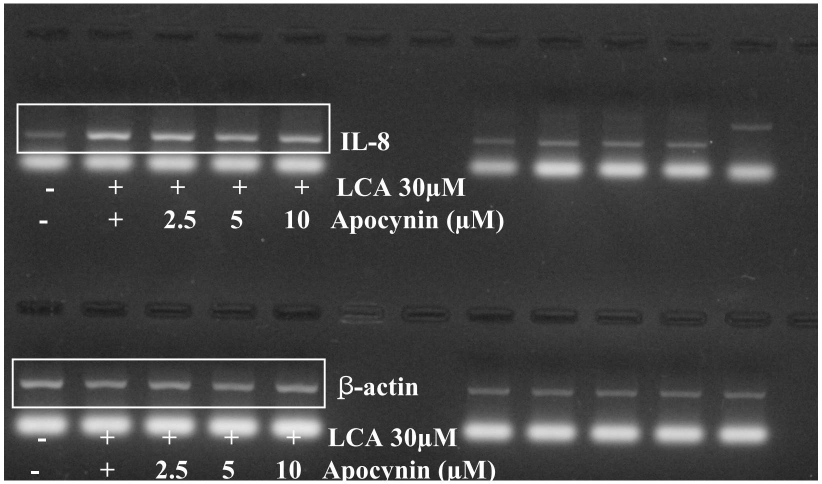

Full-length agarose gels presented in figure 5C in the article
