# Supplementary material for: Metformin inhibits lithocholic acid-induced interleukin 8 upregulation in colorectal cancer cells by suppressing ROS production and NF-kB activity
Source: Sci Rep. 2019 Feb 14;9:2003. doi: 10.1038/s41598-019-38778-2 (PMC6376015; doi:10.1038/s41598-019-38778-2)

Full-length gels and blots

Metformin inhibits lithocholic acid-induced interleukin 8 upregulation in colorectal cancer cells by suppressing ROS production and NF-kB activity

Thi Thinh Nguyen[1*], Trong Thuan Ung[1*], Shinan Li[1], Sen Lian[2], Yong Xia[3], Sun Young Park[1] and Young Do Jung[1**]

[1]Department of Biochemistry, Chonnam National University Medical School, Seoyang Ro 264, Hwasun, Jeonnam 58138, Korea

[2]Department of Biochemistry and Molecular Biology, School of Basic Medical Sciences, Southern Medical University, Guangzhou, 510515, Guangdong, China

[3]Department of Urology, New York University School of Medicine, New York, NY, 10010, USA

****Corresponding author:** Young Do Jung, MD., PhD.

Department of Biochemistry, Chonnam National University Medical School, Seoyang Ro 264, Hwasun, Jeonnam 58138, Korea; Telephone: 82-61-379-2772, Fax: 82-81-379-2781, Email: ydjung@chonnam.ac.kr

* These authors contributed equally to this work

Full-length scans of the western blots presented in Figure 2A in the main text

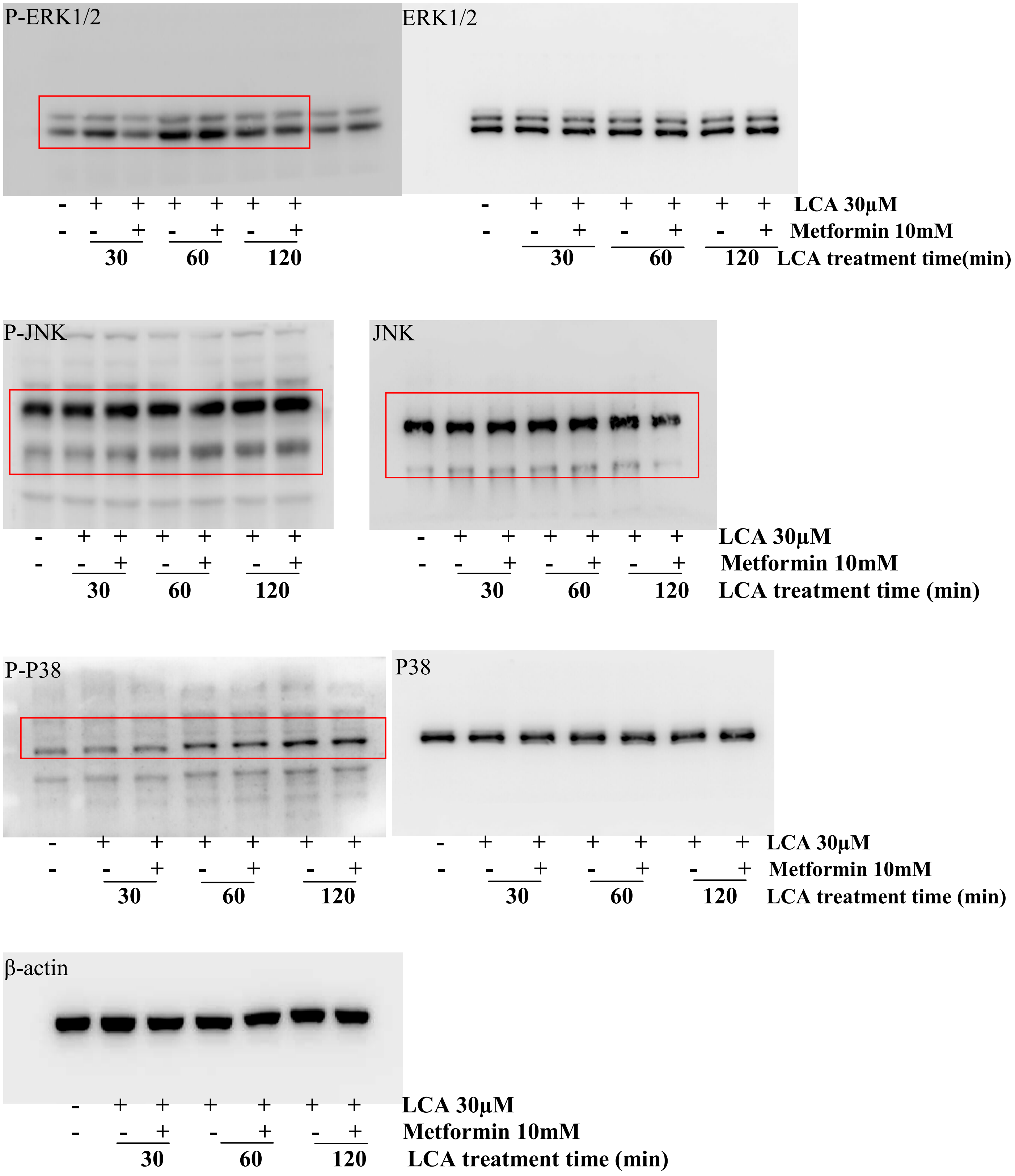

Full-length scans of the blots presented in Figure 2D in the main text

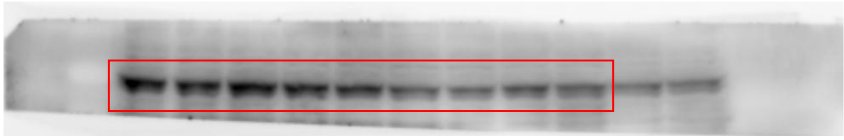

P-STAT3

- + + + + + + + + LCA 30μM
- - + - + - + - + Metformin 10mM
30 60 120 240 LCA treatment time (min)

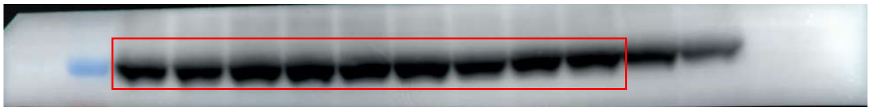

STAT3

- + + + + + + + + LCA 30μM
- - + - + - + - + Metformin 10mM
30 60 120 240 LCA treatment time (min)

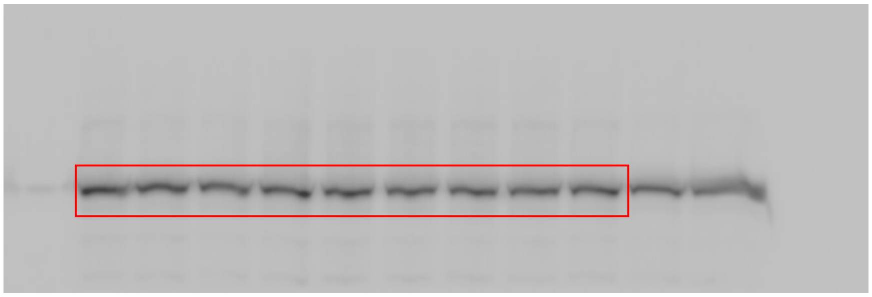

β-actin

- + + + + + + + + LCA 30μM
- - + - + - + - + Metformin 10mM
30 60 120 240 LCA treatment time (min)

Full-length scans of the blots presented in Figure 3D in the main text

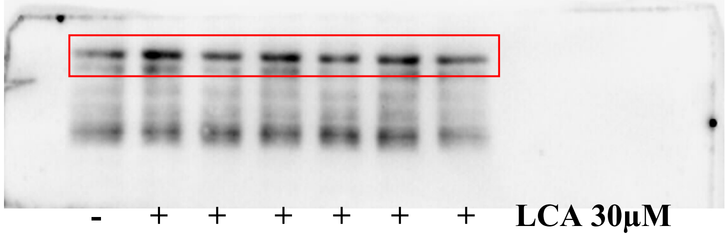

P65-nuclear

\- + + + + + + LCA 30μM

\- - + - + - + Metformin 10mM

30 60 120 LCA treatment time (min)

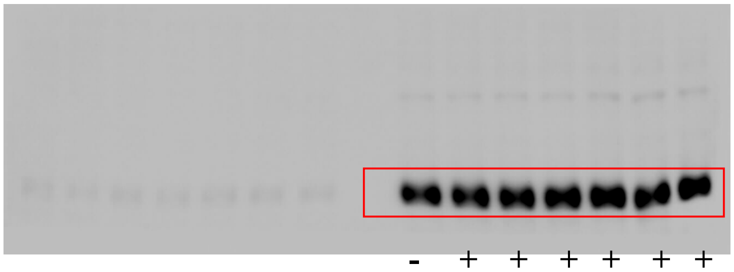

TBP-nuclear

\- + + + + + + LCA 30μM

\- - + - + - + Metformin 10mM

30 60 120 LCA treatment time (min)

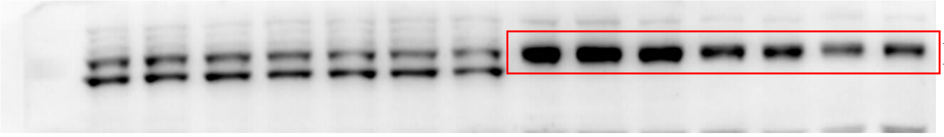

P65-cytoplasm

\- + + + + + + LCA 30μM

\- - + - + - + Metformin 10mM

30 60 120 LCA treatment time (min)

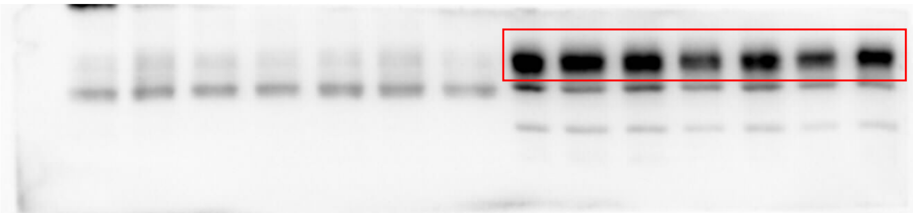

IkBα-cytoplasm

\- + + + + + + LCA 30μM

\- - + - + - + Metformin 10mM

30 60 120 LCA treatment time (min)

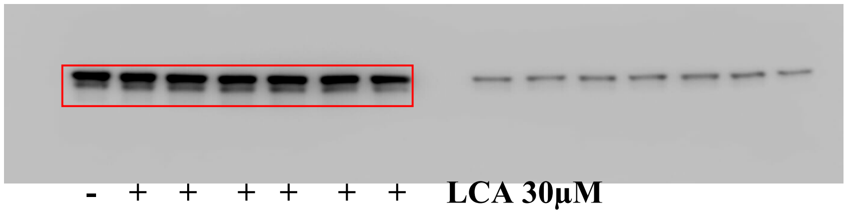

β-actin cytoplasm

\- + + + + + + LCA 30μM

\- - + - + - + Metformin 10mM

30 60 120 LCA treatment time (min)

Agarose gels presented in Figure 1 in the main text

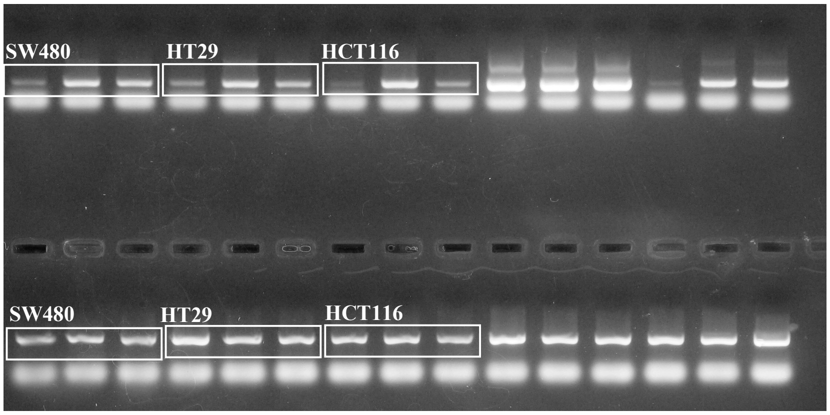

IL-8

β-actin

Figure 1A

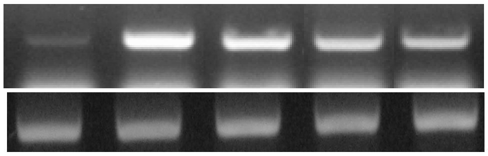

IL-8

β-actin

Figure 1B

**-**	+	+	+	+	**LCA 30µM**
**-**	**-**	**5**	**10**	**20**	**Metformin (mM)**

Agarose gels presented in Figure 3 in the main text

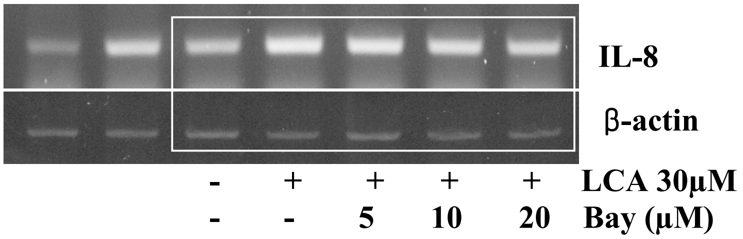

Figure 3A

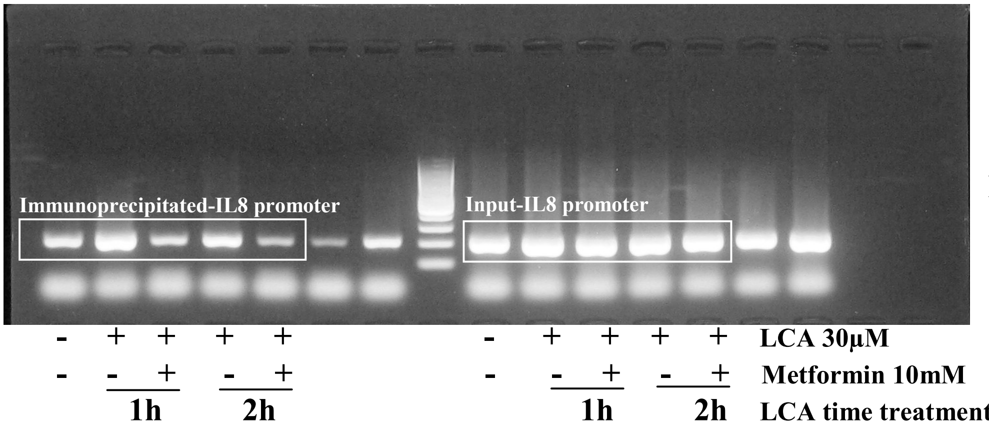

Figure 3E

Agarose gels presented in Figure 5C in the main text

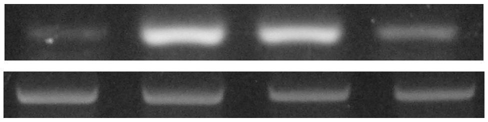

IL-8

β-actin

-	+	+	+	LCA 30μM
-	-	15	30	DPI (μM)

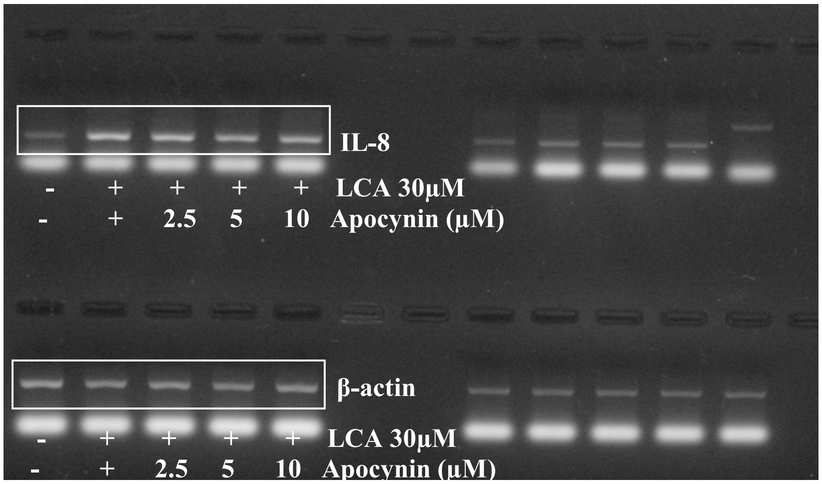

Supplement: Supplementary file 2 — Full-length gels and blots [file 41598_2019_38778_MOESM2_ESM.pdf]
